# Genetic Interaction of tRNA-Dependent Mistranslation with Fused in Sarcoma Protein Aggregates

**DOI:** 10.3390/genes14020518

**Published:** 2023-02-18

**Authors:** Jeremy T. Lant, Farah Hasan, Julia Briggs, Ilka U. Heinemann, Patrick O’Donoghue

**Affiliations:** 1 Department of Biochemistry, The University of Western Ontario, London, ON N6A 5C1, Canada; 2 Department of Chemistry, The University of Western Ontario, London, ON N6A 5C1, Canada

**Keywords:** amyotrophic lateral sclerosis (ALS), fused in sarcoma (FUS) protein, mistranslation, protein aggregation, protein synthesis, transfer RNA (tRNA)

## Abstract

High-fidelity protein synthesis requires properly aminoacylated transfer RNAs (tRNAs), yet diverse cell types, from bacteria to humans, show a surprising ability to tolerate errors in translation resulting from mutations in tRNAs, aminoacyl-tRNA synthetases, and other components of protein synthesis. Recently, we characterized a tRNA^Ser^_AGA_ G35A mutant (tRNA^Ser^_AAA_) that occurs in 2% of the human population. The mutant tRNA decodes phenylalanine codons with serine, inhibits protein synthesis, and is defective in protein and aggregate degradation. Here, we used cell culture models to test our hypothesis that tRNA-dependent mistranslation will exacerbate toxicity caused by amyotrophic lateral sclerosis (ALS)-associated protein aggregation. Relative to wild-type tRNA, we found cells expressing tRNA^Ser^_AAA_ showed slower but effective aggregation of the fused in sarcoma (FUS) protein. Despite reduced levels in mistranslating cells, wild-type FUS aggregates showed similar toxicity in mistranslating cells and normal cells. The aggregation kinetics of the ALS-causative FUS R521C variant were distinct and more toxic in mistranslating cells, where rapid FUS aggregation caused cells to rupture. We observed synthetic toxicity in neuroblastoma cells co-expressing the mistranslating tRNA mutant and the ALS-causative FUS R521C variant. Our data demonstrate that a naturally occurring human tRNA variant enhances cellular toxicity associated with a known causative allele for neurodegenerative disease.

## 1. Introduction

High-fidelity protein synthesis depends on the accurate translation of the genetic code and is considered essential for cell viability. Transfer RNAs (tRNAs) play a critical role in the translation of messenger RNAs (mRNAs) into proteins. Although errors in protein synthesis normally occur rarely, with estimates suggesting that 1 in every 1,000 to 10,000 codons is misread [1], cells can survive or tolerate significantly elevated levels of mistranslation of 1–10% per codon [2,3,4]. Thus, in the absence of other defects, cells have robust capabilities to maintain protein homeostasis and resist the error catastrophe that Orgel [5] envisioned would result from translation with reduced fidelity. 

Mistranslation results from mutations in tRNAs [6] or the aminoacyl-tRNA synthetases (AARSs) [7,8] that are responsible for ligating each tRNA with its cognate amino acid [9]. Mutations to the ribosome [10], ribosomal proteins [11], and other [12] components of the protein quality control machinery can also increase error rates in protein synthesis. Mutations in the tRNA anticodon or identity element nucleotides that each AARS uses to recognize its cognate tRNA can cause defective or mis-aminoacylation of tRNAs [13,14]. In most cases, anticodon mutations reduce or prevent tRNA aminoacylation [15]. Mutations to the anticodon in alanine (Ala), serine (Ser), and in part leucine (Leu) tRNAs, however, fail to AARS recognition, allowing the potential for amino acid mis-incorporation at different codons [3,4,14,16,17]. In a distinct mechanism, mutations to tRNA identity elements that inhibit AARS activity may cause loss-of-function [18], while mutations that enable a tRNA to acquire a new identity [6,14] can lead to the mis-aminoacylation of tRNAs and mistranslation [3,16]. 

There are over 600 tRNA genes in the human genome. In comparison to the reference genome, individuals carry many single-nucleotide [19,20] and even multi-site tRNA variants [21]. In our targeted tRNA gene sequencing study of 84 individuals, we discovered unprecedented variation in tRNA genes. The data showed that individuals harbor 60–70 single-nucleotide polymorphisms on average in their tRNA genes, including different kinds of mistranslating tRNAs that occur as both rare or more common variants in the population [21]. 

In the tRNA Ser-AGA-2-3 gene (tRNA^Ser^_AGA_), a G35A variant occurs with an allele frequency of 2–3% in the human population [6,21]. The resulting tRNA^Ser^_AAA_ mutant contains a G-to-A substitution in the second position of the anticodon. Since seryl-tRNA synthetase (SerRS) does not recognize the anticodon of tRNA^Ser^ [22], the mutant was expected to readily accept serine. Indeed, we found that the tRNA^Ser^_AAA_ variant causes the mis-incorporation of serine at phenylalanine codons in mammalian cells [17]. The mistranslating tRNA^Ser^_AAA_ also enhanced cytotoxicity and inhibited protein synthesis in cells. We previously investigated genetic interactions between mistranslation and poly-glutamine (polyQ)-expanded huntingtin alleles. Although cells expressing the tRNA^Ser^ G35A variant were slow but effective in forming huntingtin aggregates, mistranslating cells were defective in degrading and clearing disease-causing polyQ aggregates. The data suggested that natural tRNA variants compromise translation fidelity with the potential to affect both the age of onset and severity of Huntington’s disease [17]. 

Amyotrophic lateral sclerosis (ALS) is a neurodegenerative disease caused by the aggregation of proteins in neurons, leading to muscle wasting, weakness, and eventual death [23]. The aggregation of nuclear RNA binding proteins, notably the fused in sarcoma (FUS) protein, is commonly associated with ALS [24]. FUS is a DNA and RNA binding protein that regulates transcription and mRNA processing in neurons [25]. There are 19 known FUS mutations that are linked to the development of ALS, and FUS mutations are found in >4% of patients who present with the disease [26,27,28,29]. Most causative mutations are in the C-terminal nuclear localization signal (NLS) domain, which partially prevents the translocation of FUS into the nucleus, where it normally regulates DNA replication kinetics [30], RNA splicing [31,32], and transcription through interactions with RNA polymerase II, transcription factor TFIID, and directly with RNA [33,34]. Neurodegeneration linked to FUS mutations may arise from both loss of its nuclear function and the gain of toxic function in the cytosol [35]. While some ALS mutants in the FUS NLS domain cause ~50% of the protein to mis-localize to the cytoplasm, the FUS Arg521Cys (R521C) mutation causes aggregate formation [36] and a minor nuclear trafficking defect with only ~10% of the protein mis-localized to the cytoplasm [37]. Individuals carrying mutations such as R521C tend to develop early-onset ALS, with 60% of cases occurring before 40 years of age [32]. 

To establish the potential for mistranslating tRNAs to modify neurogenerative disease-associated protein aggregation, we employed cellular models of FUS protein aggregation [38,39]. We found that mistranslating cells readily formed wild-type FUS protein aggregates but at a slower rate compared to cells expressing wild-type tRNA. In cells expressing the ALS-causative FUS allele, mistranslation caused synthetic toxicity because of rapid FUS aggregate formation that led to cell rupture events. The data show that naturally occurring mistranslating tRNAs increase cytotoxicity and modify protein aggregation kinetics of FUS alleles in cells. 

## 2. Materials and Methods

### 2.1. Plasmids and Strains 

Plasmid manipulations were performed in *Escherichia coli* DH5α cells (Invitrogen Canada, Burlington, ON, Canada). The wild-type tRNA^Pro^ and G3:U70 variants were expressed from a U6 promoter with a polythymidine terminator, as previously described [3]. The human tRNA^Ser^ gene (Ser-AGA-2-3) was polymerase chain reaction (PCR)-amplified from human embryonic kidney (HEK) 293T cell genomic DNA with ±300 bp of native flanking sequence. The G35A anticodon variants were introduced in PCR fragments using overlap extension PCR, as previously described [17]. The tRNA expression cassettes were inserted at the *Pci*I restriction site in pWTPAN-derived plasmids or at the *Nru*I site in pcDNA3.1-derived plasmids. Fusions of the FUS gene with fluorescent proteins were created by PCR amplifying the full-length FUS gene (encoding residues 1–526) from a pcDNA3.1 plasmid containing the human FUS gene isoform 1 (a kind gift of Dr. Michael Strong) with a primer-encoded flexible linker sequence (amino acid sequence GGGSGG). EGFP fusions were created by inserting the FUS-linker sequence into *Nhe*I and *Hind*III restriction sites in our previously described pcDNA3.1-EGFP plasmid [3]. FUS-mCherry fusion constructs were also created by inserting the FUS-linker PCR product into the *Nhe*I and *Spe*I sites of WT-PAN [40]. WT-PAN contains an EGFP-mCherry fusion protein, and our approach replaced the EGFP segment with the FUS gene and linker sequence to create FUS-mCherry fusions. The R521C variant of the FUS-mCherry protein was created by round-the-horn PCR mutagenesis using primers (Table S1), including the mutant nucleotide C1561T. Plasmids containing mCherry were created by digesting and cross-ligating the isoschizomeric *Nhe*I and *Spe*I sites in WT-PAN to remove the EGFP gene. 

### 2.2. Cell Culture and Transfections 

Experiments were performed in murine Neuro2a neuroblastoma (N2a) cells (American Type Culture Collection, ATCC #CCL-131) or HEK 293T cells (ATCC #CRL-3216). All cell lines were grown at 37 °C with humidity and 5% CO_2_. Cells were cultured in high glucose Dulbecco’s modified Eagle medium (DMEM with 4.5 g/L glucose, Gibco by Life Technologies, Carlsbad, CA, USA) containing penicillin (100 IU/mL, Wisent Bioproducts, Montreal, QC, Canada), streptomycin (100 µg/mL, Wisent Bioproducts, Saint-Jean-Baptiste, QC, Canada), and 10% fetal bovine serum (FBS, Gibco). All transfections were performed using Lipofectamine 3000 transfection reagent (Invitrogen, ThermoFisher, Ottawa, ON, Canada) with 2 µg/mL plasmid DNA, following the manufacturer’s instructions. 

Details concerning cell harvesting, Western blotting, cytotoxicity assays, and statistical analysis are included in the Supplementary Information. 

### 2.3. tRNA Sequencing

N2a cells were transfected for 48 h in biological triplicates on 10 cm plates with plasmids encoding tRNA^Ser^_AGA_ (Ser-AGA-2-3 gene) or the tRNA^Ser^_AAA_ (Ser-AAA-2-3 gene) variant and mCherry as a transfection marker (Figure S1). We estimated transfection efficiency by counting the number of visibly fluorescing cells in images from each transfection. Based on this count, we estimated ~60% transfection efficiency. Cells were harvested by resuspension in TRIzol reagent (ThermoFisher, Ottawa, ON, Canada), then stored in liquid nitrogen before sending to Arraystar, Inc. (Rockville, MD, USA) for tRNA sequencing. Details of sample preparation and the tRNA sequencing methods are provided in the Supplementary Information. 

### 2.4. Fluorescence Microscopy

Microscopy images were captured on an EVOS FL Auto 2 imaging system (ThermoFisher) with brightfield and fluorescence imaging using GFP (470 ± 22 nm excitation, 510 ± 42 nm emission) or RFP (531 ± 40 nm excitation, 593 ± 40 nm emission) filter cubes. All images were captured with the EVOS 4× objective (fluorite, PH, long-working distance, 0.13 numerical aperture/10.58 mm working distance). We refined standard aggregation analysis by adding a median size adjustment step, where cellular area and aggregate area were normalized to their respective median area values across the entire experiment (see Supplemental Information). Aggregates of FUS begin to form at 24 h post-transfection, and all images of cells were taken between 24 and 72 h post-transfection. For live cell imaging, cells were incubated in the EVOS environment chamber at 37 °C and 5% CO_2_ with humidity. Images were captured every 30 min beginning 24 h after the start of transfection for time courses of 18 to 44 h as indicated. Before beginning the time course, culture plates were placed in the environment chamber for 1 h to acclimate before fine-tuning fields of view. Fluorescence per cell and number of aggregates per cell were quantitated using a semi-automated approach in ImageJ (see Supplementary Information). 

### 2.5. Semi-Denaturing Detergent Agarose Gel Electrophoresis (SDD-AGE)

Cell lysates were prepared, and protein concentrations were measured 72 h post-transfection as described above. A 1.5% agarose gel and Tris-acetate ethylenediaminetetraacetic acid (EDTA) (TAE) running buffer containing 40 mM Tris-acetate, 1 mM EDTA, and 0.1% *w*/*v* sodium dodecyl sulfate (SDS) were prepared according to established protocols [41]. Lysate samples containing 20 μg of protein were diluted in 3× loading dye (0.5 M Tris-HCl, pH 6.8, 1.12 M sucrose; 0.025% *w*/*v* bromophenol blue; 3.8% *w*/*v* SDS) with sterile double-distilled H_2_O. Lysates were separated on the agarose-SDS gel for at least 3 h at 20 V. Proteins were transferred to a polyvinylidene difluoride (PVDF) membrane by capillary gel transfer overnight using TAE with 0.1% SDS as a buffer. mCherry-tagged FUS aggregates were visualized by Western blotting with an α-mCherry antibody.

## 3. Results

### 3.1. Protein Production in Mistranslating Cells

**Figure 1:**
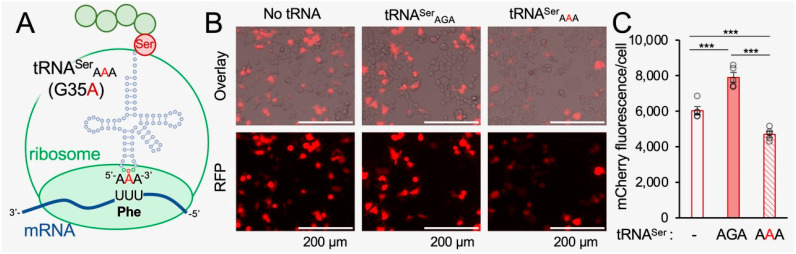
mCherry production in neuroblastoma cells expressing wild-type or mistranslating tRNAs. (**A**) The human tRNA^Ser^_AGA_ G35A mutation creates a tRNA^Ser^_AAA_ variant that mistranslates Phe codons with Ser in mammalian cells [17]. N2a cells were transfected with a plasmid bearing no additional tRNA, tRNA^Ser^_AGA_, or tRNA^Ser^_AAA_ and mCherry. (**B**) Images of fluorescing cells (bright field overlay, top) were captured by live-cell fluorescence microscopy (RFP; ex. 531 nm, em. 593 nm, bottom) at 24 h after transfection. (**C**) The mCherry fluorescence per cell was quantitated (see Supplementary Appendix). Error bars represent the mean ± 1 standard deviation of at least four biological replicates. Significant differences from pairwise independent sample *t*-tests are indicated (*** *p* < 0.001).

The human tRNA Ser-AGA-2-3 gene has a mistranslating anticodon variant found in ~2% of sequenced individuals [6,17,20]. The G35A variant converts the Ser-decoding AGA anticodon to a Phe-decoding AAA anticodon [6,17]. We cloned the tRNA Ser-AGA-2-3 gene and the G35A mistranslating variant (Figure 1A) with ±300 bps of native sequence context into plasmids that co-express mCherry, which serves as a transfection marker and reporter for protein production. We and others have shown that fluorescent proteins are appropriate markers for protein levels in normal and mistranslating cells [4,17,42]. In agreement with our previous observations, co-expression of mCherry with the wild-type tRNA^Ser^ in murine neuroblastoma (N2a) cells caused a significant 33% increase in mCherry fluorescence per cell. Interestingly, the tRNA^Ser^_AAA_ mutant caused a significant 22% reduction in mCherry fluorescence per cell compared to cells expressing mCherry with no ectopic tRNA (Figure 1B,C), demonstrating that tRNA^Ser^_AAA_ causes a dominant negative defect in mCherry fluorescence in mammalian cells.

We previously demonstrated that N2a cells expressing tRNA^Ser^_AAA_ show reduced protein production and reduced rates of protein degradation compared to N2a cells expressing the wild-type tRNA [17]. Here (see Section 3.5) and previously [17], we established that the reduction in mCherry fluorescence per cell correlates with reduced mCherry protein levels according to Western blotting in N2a cells. We also used liquid chromatography combined with tandem mass spectrometry (LC-MS/MS) to confirm that expression of tRNA^Ser^_AAA_ leads to significant mis-incorporation of Ser at Phe codons [17]. Based on our previous LC-MS/MS data, spectral counting provided an approximate estimate that tRNA^Ser^_AAA_ produced serine mis-incorporation at a level of ~9% at the Phe codons of mCherry produced in mistranslating N2a cells [17] and that expression of tRNA^Ser^_AAA_ causes a dominant negative phenotype, leading to reduced protein abundance in mammalian cells. 

### 3.2. tRNA Sequencing Identifies and Quantitates tRNA^Ser^_AAA_ Abundance in Mistranslating Cells

To verify the expression and quantify the abundance of the tRNA^Ser^ mutant, we performed tRNA sequencing. The Hydra-Seq method (see Supplementary Methods) was used to measure read counts for all tRNA transcripts in N2a cells transfected with a plasmid expressing the wild-type tRNA^Ser^_AGA_ or the mistranslating tRNA^Ser^_AAA_ in three biological replicates each (see Figure S1, Supplementary Data File S1). In comparing raw read counts of all tRNAs in wild-type or mistranslating N2a cells, we observed that no tRNAs were significantly changed in abundance by 2-fold or more (Figure S2A). Only two tRNAs were significantly increased in abundance by 1.5-fold or more; tRNA^Lys^_UUU_ (Lys-TTT-1-1) was upregulated by 1.8-fold, and the mitochondrial tRNA^Cys^_CGA_ was upregulated by 1.5-fold. We also normalized the read counts by the total number of reads in each sample. A volcano plot based on the normalized read counts shows that no tRNAs were significantly changed by 2-fold or more (Figure S2B). The normalized read count data also indicated that tRNA^Lys^_UUU_ was significantly and slightly increased in abundance (1.7-fold) in wild-type compared to mistranslating cells. The data suggest that, overall, the tRNA pools are very similar between wild-type and mistranslating cells. 

**Figure 2:**
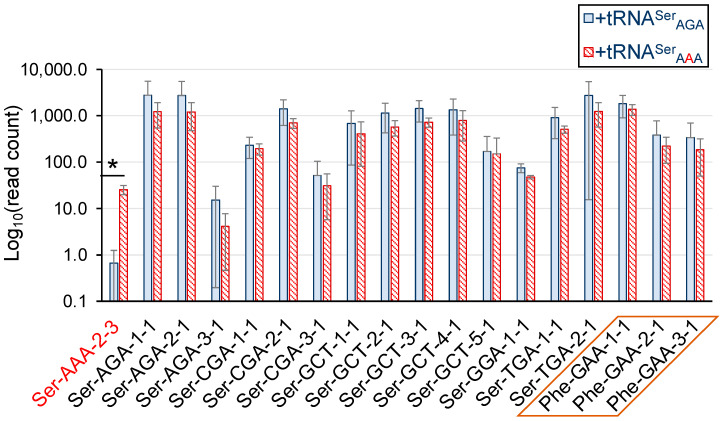
Quantitation of tRNA levels in N2a cells expressing wild-type or mistranslating tRNAs. In N2a cells transfected with a plasmid bearing wild-type tRNA^Ser^_AGA_ (blue bars) or the mistranslating tRNA^Ser^_AAA_ variant (red hashed bars), the abundance of all tRNA transcripts was quantified using the Hydra-seq method of tRNA sequencing (see Supplementary Information, Figures S1 and S2). The abundance of tRNA^Ser^ and tRNA^Phe^ isoacceptors was plotted as the Log_10_ of read counts. As anticipated, there is a significant abundance of tRNA^Ser^_AAA_ detected only in cells expressing the mistranslating tRNAs. On average, we observed less than 1 read count on average for tRNA^Ser^_AAA_ in cells transfected with a plasmid expressing wild-type tRNA^Ser^, while in cells transfected with a plasmid expressing the mistranslating tRNA, we observed ~26 read counts on average for tRNA^Ser^_AAA_. The abundance of other Ser-decoding tRNAs or Phe-decoding tRNAs (orange box) was not changed significantly in mistranslating cells. Error bars represent the mean ± 1 standard deviation of three biological replicates. Significant differences from pairwise independent sample *t*-tests are indicated (* *p* < 0.05); all other pairwise comparisons between wild-type and mistranslating cells were not significant.

The tRNA sequencing data confidently identified the expression of tRNA^Ser^_AAA_ only in cells transfected with the plasmid bearing the Ser-AAA-2-3 allele. We observed <1 read count on average for tRNA^Ser^_AAA_ in cells expressing the wild-type tRNA^Ser^, indicating a minimal background level of sequencing error. In cells expressing the tRNA^Ser^_AAA_ allele, we observed ~26 read counts on average across the biological replicates (Figure 2). Because of the unique mutation in this tRNA gene, read counts for the mutant tRNA are found only among the unique mapped reads (Supplementary Data File S1). The tRNA^Ser^_AGA_ pool is produced in cells from 6 identical gene copies (Ser-AGA-2-1, Ser-AGA-2-2, Ser-AGA-2-3, Ser-AGA-2-4, Ser-AGA-2-5, and Ser-AGA-2-6). Because tRNA sequencing measures the level of the mature tRNA, the method cannot disambiguate the contributions to the tRNA^Ser^_AGA_ pool from each of the identical Ser-AGA genes. Thus, read count data for the tRNA^Ser^_AGA_ pool are identified only among the multi-mapped reads (Supplementary Data File S1). Our data show that, together, the 6 tRNA^Ser^_AGA_ genes yield a tRNA pool of ~2000 read counts on average. Statistical analysis showed that the level of the tRNA^Ser^_AGA_ pool is indistinguishable in cells expressing either the plasmid-borne Ser-AGA-2-3 gene or the mutant Ser-AAA-2-3 gene (Figure 2). 

In mistranslating cells, the tRNA^Ser^_AAA_ makes up 0.3% of the total tRNA^Ser^ pool (Figure 2) and 2.1% of the tRNA^Ser^_AGA_ pool. The mistranslating tRNA^Ser^_AAA_ does not compete with the tRNA^Ser^ molecules in translation as it only decodes Phe codons. In comparison to the levels of the tRNA^Phe^_GAA_ pool that decode the Phe UUU/C codons, tRNA^Ser^_AAA_ makes up 1.4% of Phe decoders. Factoring in our transfection efficiency of 60% (Figure S1), the level of tRNA^Ser^_AAA_ was approximately 3% of Phe decoders. Our tRNA sequencing data demonstrate that the tRNA^Ser^_AAA_ is expressed at physiological levels without substantially altering the overall abundance of other tRNAs in the cell. 

The level of tRNA^Ser^_AAA_ is similar to and slightly less than the ~9% level of Ser mis-incorporation at Phe codons we estimated previously according to mass spectrometry [17]. The tRNA expression level is only one of several factors that impact the decoding efficiency of a particular aminoacyl-tRNA on the ribosome. Aminoacylation level, codon-anticodon pairing, and competition with other aminoacyl-tRNAs for the same codon all impact the level of mistranslation. For example, there are no native Phe decoders with the AAA anti-codon, which may enable Ser-tRNA^Ser^_AAA_ to efficiently compete against Phe-tRNA^Phe^_GAA_ in decoding the Phe (UUU/C) codons. 

### 3.3. Aggregation of FUS Alleles in Normal and Mistranslating Cells

**Figure 3:**
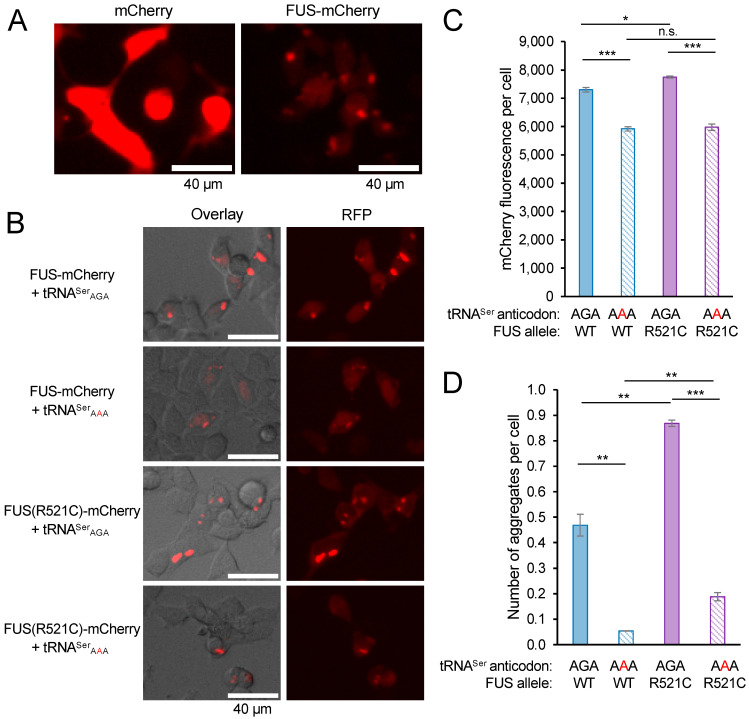
FUS-mCherry aggregates in N2a cells and in HEK 293T cells expressing wild-type or mistranslating tRNA. (**A**) N2a cells expressing mCherry (and no additional tRNA) show diffuse fluorescence well-distributed in the cells, while the cells expressing FUS-mCherry (and no additional tRNA) show dimmer and diffuse fluorescence throughout the cells, as well as clearly defined foci representing FUS aggregates. (**B**) At 24 h after transfection, representative images of HEK 293T cells co-expressing human tRNA^Ser^_AGA_ or G35A variant (tRNA^Ser^_AAA_) and FUS-mCherry or FUS R521C-mCherry were captured by brightfield imaging overlayed (left) with fluorescence microscopy imaging (RFP; ex. 531 nm, em. 593 nm, right); the FUS-mCherry fluorescence per cell (**C**) and the number of FUS aggregates per cell (**D**) were quantified (see Supplementary Information). Error bars represent the mean ± 1 standard deviation of at least three biological replicates. Significant differences from pairwise independent sample *t*-tests are indicated (n.s.—not significant, * *p* < 0.05, ** *p* < 0.01, *** *p* < 0.001).

To monitor the synthesis of FUS proteins in mistranslating cells, we cloned wild-type FUS and FUS R521C with C-terminal mCherry tags that were expressed from plasmids containing wild-type tRNA^Ser^ or the mistranslating tRNA^Ser^_AAA_ [6,17]. FUS aggregation in live cells has been investigated using fluorescent protein fusions to the C-terminal [36,38,39,43,44], N-terminal [45,46], and even both termini [47] to provide a fluorescent sensor for FUS conformational dynamics. C-terminally tagged FUS-GFP predominantly localizes to the nucleus [38] and provides an appropriate model for FUS protein aggregation in cells [36,38,39,43,44]. Because FUS R521C has a minor and well-characterized localization defect [37], we focused our studies on the kinetics of FUS aggregation in live cells and in the context of reduced translation fidelity. We transfected N2a cells, a well-established cell line for studies of FUS aggregation [48], with a plasmid expressing mCherry or FUS-mCherry with no additional tRNA (Figure 3A). In contrast to N2a cells expressing mCherry, where fluorescence is well distributed throughout the cell, cells expressing FUS-mCherry show dimmer diffuse fluorescence in the cell with clearly defined foci representing FUS aggregates. 

To demonstrate FUS aggregation in an independent cell line, we also transfected HEK 293T cells with plasmids bearing FUS-mCherry or FUS R521C-mCherry and wild-type tRNA^Ser^_AGA_ or the tRNA^Ser^_AAA_ variant (Figure 3B). We observed the production of both FUS-mCherry variants in normal and mistranslating cells at 24 h post-transfection. We identified foci in cells expressing either of the FUS-mCherry alleles, demonstrating the formation of subcellular FUS aggregates (Figure 3B). The data confirm the formation of FUS and FUS R521C aggregates in cells expressing wild-type tRNA^Ser^, as well as the mistranslating tRNA mutant. Consistent with our previous study [17], we observed significantly reduced FUS-mCherry fluorescence levels in cells expressing the mutant tRNA (Figure 3C). 

We used a previously developed thresholding method [17] (see Supporting Information) to automatically identify the intense fluorescent foci that represent FUS aggregates. Our approach is similar to other well-established methods to automate protein aggregate counting in cells [49]. We used a stringent fluorescence intensity threshold equivalent to 18 standard deviations above the average FUS-mCherry fluorescence per cell to identify bright foci that represent protein aggregates. The method provides a quantitation of the number of aggregates forming in each transfected cell (Figure 3D). In both normal and mistranslating HEK 293T cells, we observed significantly more FUS R521C aggregates per cell compared to wild-type FUS aggregates (Figure 3D). For both the wild-type and mutant FUS, mistranslating cells formed a reduced number of FUS aggregates per cell compared to cells expressing the wild-type tRNA. 

### 3.4. Toxicity of FUS Alleles in Mistranslating Cells

**Figure 4:**
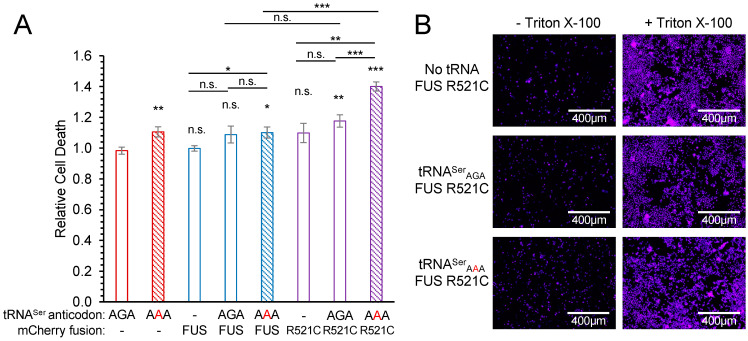
Synthetic toxicity of mistranslation and FUS R521C aggregation in N2a cells. N2a cells were transfected with a plasmid encoding no additional tRNA, human tRNA^Ser^_AGA_, or G35A variant (tRNA^Ser^_AAA_) and mCherry, FUS-mCherry, or FUS R521C-mCherry. At 72 h post-transfection, cytotoxicity was assayed by determining relative levels of dead cells using Sytox blue dye and the ratio of fluorescence before (dead cells) and after (total cells) treatment with a cell membrane detergent (Triton X-100). (**A**) Biological means were normalized to the mean of the wild-type tRNA and mCherry control. Error bars represent the mean ± 1 standard deviation of five biological replicates. Significant differences from pairwise independent sample *t*-tests are indicated (n.s.—not significant, * *p* < 0.05, ** *p* < 0.01, *** *p* < 0.001). Annotations above each bar represent pairwise *t*-tests with AGA/mCherry; other pairwise *t*-test are indicated. (**B**) Representative images (see also Figure S3) were captured by fluorescence microscopy (CFP; ex. 445 nm, em. 510 nm).

To assess cytotoxicity from the genetic interactions of tRNA and FUS variants, we used a fluorescence-based dye exclusion assay [50,51]. In cells expressing only mCherry, mistranslation resulting from tRNA^Ser^_AAA_ alone led to a small but significant ~10% increase in cell death (*p* < 0.01) (Figure 4). Similarly, mistranslating cells expressing wild-type FUS-mCherry showed a significant (*p* < 0.05) but small increase in cytotoxicity compared to cells with no additional tRNA and wild-type FUS. These data indicate that mistranslation leads to a mild increase in cell death.

Cells co-expressing FUS R521C and the wild-type tRNA^Ser^_AGA_ showed a significant but moderate 17% increase in cell death compared to cells expressing mCherry with the wild-type tRNA^Ser^_AGA_. Although cells expressing wild-type tRNA were not significantly more toxic when expressing FUS R521C compared to FUS, mistranslating cells were significantly more toxic when expressing FUS R521C. Strikingly, we observed super-additive or synthetic toxicity in cells expressing both FUS R521C and the mistranslating tRNA. Compared to any other condition tested, we quantified the most significant increase in cell death in mistranslating cells that also expressed the FUS R521C allele (Figure 4 and Figure S3). The ratio of dead cell to total cell fluorescence was 40% higher in cells expressing the mutant tRNA and mutant FUS compared to cells expressing wild-type FUS with no additional tRNA. Mistranslating cells expressing the mutant FUS also showed a significant 33% increase in cell death compared to cells with no additional tRNA and mutant FUS and a 28% increase in cell death compared to cells co-expressing wild-type tRNA and mutant FUS. Our data represent the first report of a synthetic toxic genetic interaction between mistranslation caused by a natural human tRNA variant and cell death induced by an ALS-associated FUS allele. 

### 3.5. Kinetics of FUS Protein Production in Normal and Mistranslating Cells

**Figure 5:**
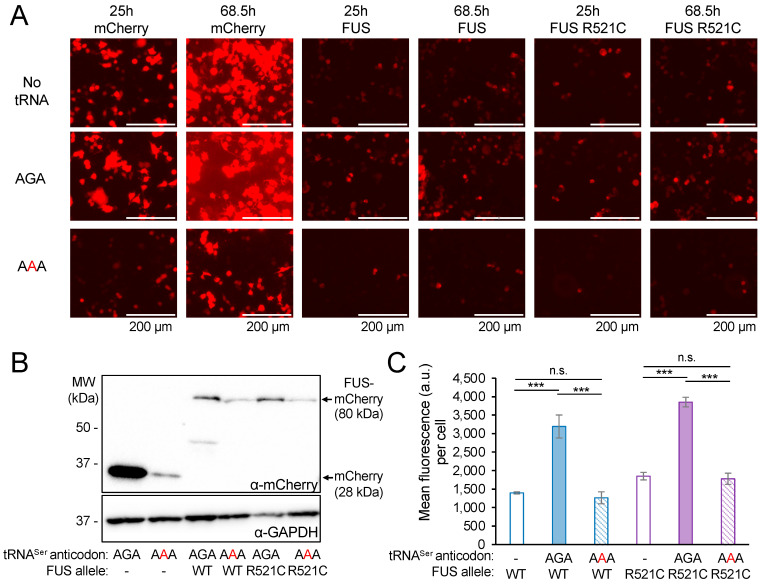
Total mCherry and FUS-mCherry protein levels in N2a cells co-expressing wild-type or mistranslating tRNA. N2a cells were transfected with a plasmid encoding no tRNA, tRNA^Ser^_AGA_ or G35A variant (tRNA^Ser^_AAA_) and mCherry, human FUS-mCherry, or FUS R521C-mCherry. Images of fluorescing cells were captured by live-cell fluorescence microscopy (RFP; ex. 531 nm, em. 593 nm) beginning 24 h after transfection for a 43.5 h time lapse (see Figure S4). (**A**) Representative images at the start (25 h) and end (68.5 h) of the time-lapse are shown. (**B**) Western blotting was performed to monitor total mCherry and FUS-mCherry protein levels in N2a cells co-expressing wild-type tRNA^Ser^ or the mistranslating tRNA^Ser^_AAA_ mutant using antibodies specific for mCherry and GAPDH as a loading control. (**C**) Total FUS-mCherry and FUS R521C-mCherry levels are represented by the mean mCherry fluorescence per cell observed at the final (68.5 h) time point. Error bars represent the mean ± 1 standard deviation of at least five biological replicates. Significant differences from pairwise independent sample *t*-tests are indicated (n.s.—not significant, *** *p* < 0.001).

To monitor the kinetics of FUS protein production, we used live-cell imaging to capture the fluorescence of FUS-mCherry or FUS R521C-mCherry proteins in individual cells over a 43.5 h time-course beginning 25 h post-transfection. In agreement with previous studies [48], we found that FUS-mCherry fluorescence was substantially lower than fluorescence from mCherry alone in cell lines expressing the same tRNA variant (Figure 5). During the time course, we observed a consistent and significant 2.3-fold increase in FUS-mCherry and a 2.0-fold increase in FUS R521C-mCherry fluorescence in cells expressing the wild-type tRNA^Ser^_AGA_ compared to cells with no additional tRNA (Figure 5C and Figure S4). We measured a similar level of FUS production in mistranslating cells compared to cells with no additional tRNA. 

In cells expressing the tRNA^Ser^_AAA_ variant, we observed fewer living cells with visible FUS-mCherry fluorescence, and the fluorescence per cell was significantly reduced by 2.5-fold for FUS-mCherry and by 2.1-fold for FUS R521C-mCherry compared to cells expressing the wild-type tRNA^Ser^_AGA_ (Figure 5C and Figure S4). Western blotting using an mCherry antibody and GAPDH loading control revealed a consistent trend in FUS protein levels (Figure 5B). Namely, the mCherry protein was produced at a higher level than the FUS fusion proteins, and mistranslating cells produce less mCherry and less FUS-mCherry protein than cells expressing the wild-type tRNA^Ser^_AGA_. 

### 3.6. FUS Aggregation Kinetics in Normal and Mistranslating Cells

Mistranslation impacts all proteins by introducing proteome-wide mutations. To assess the impact of introducing a mistranslating tRNA into N2a cells, we quantified wild-type FUS aggregate formation in N2a cells. We used the thresholding approach noted above (Section 3.3, Supplementary Information) to count the number of FUS aggregates per cell over a time course in normal N2a cells and in N2a cells with different kinds of mistranslation. We first assayed aggregation kinetics of FUS-eGFP fusion protein co-expressed with a synthetic variant of tRNA^Pro^ (G3:U70), which we previously established causes the mistranslation of proline codons with alanine but does not inhibit protein synthesis in mammalian cells [3]. Consistent with our previous findings on this tRNA and models of huntingtin polyQ proteins [17], we did not observe any significant differences in FUS-eGFP aggregation in cells expressing wild-type tRNA^Pro^ or the tRNA^Pro^ G3:U70 alanine-accepting variant (Figure S5). 

**Figure 6:**
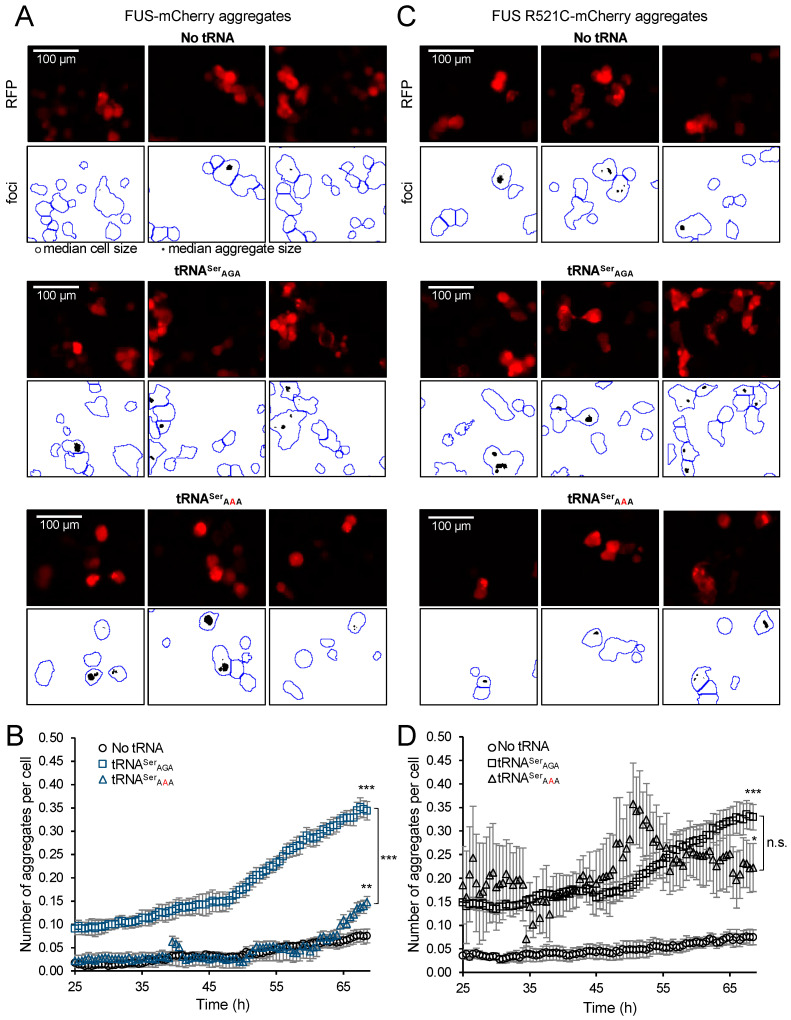
Mistranslation modifies the aggregation kinetics of FUS and FUS R521C in N2a cells. The number of FUS aggregates per cell was quantified at each time point. N2a cells were transfected with a plasmid encoding no tRNA, human tRNA^Ser^_AGA_, or tRNA^Ser^_AAA_ and (**A**,**B**) FUS-mCherry or (**C**,**D**) FUS R521C-mCherry. Representative images (see Figures S7 and S8) were captured by live-cell fluorescence microscopy (RFP ex. 531 nm, em. 593 nm) for 43.5 h time course. Fluorescent cell area representing cells and aggregates was determined using a custom Fiji/ImageJ macro (see Supplementary Information). Cell and aggregate counts were determined based on the observed median cell and aggregate area. (**A**,**C**) Representative images of the fluorescing cells and black and white mask images showing aggregate area (black) and cell area (blue outlines). (**B**,**D**) The number of aggregates per cell was plotted over time. Error bars represent the mean ± 1 standard deviation of at least four biological replicates. Significant differences (see also Figure S6) based on pairwise independent sample *t*-tests are indicated (* *p* < 0.05, ** *p* < 0.01, *** *p* < 0.001) and annotated above the final time point in comparison to the no tRNA control and between AAA/AGA by brackets.

We next assessed the aggregation kinetics of FUS-mCherry and FUS R521C-mCherry proteins in individual transfected cells co-expressing no additional tRNA, wild-type tRNA^Ser^, or the tRNA^Ser^_AAA_ variant. In cells expressing wild-type tRNA^Ser^_AGA_, the number of FUS-mCherry aggregates per cell was 5-fold increased at the start of the time course and 4-fold increased at the end of the time course compared to cells expressing no additional tRNA (Figure 6A,B and Figures S6A and S7). This indicates that the addition of a wild-type tRNA accelerates FUS-mCherry aggregate formation.

In cells expressing the tRNA^Ser^_AAA_ mutant and FUS-mCherry, the number of aggregates per cell did not differ significantly at the beginning of the time course compared to cells expressing no additional tRNA (Figure 6A,B and Figures S6A and S7). In the final 5 h of the time course, however, the number of aggregates per cell increased in cells expressing tRNA^Ser^_AAA_ and FUS-mCherry to a point where there were 2-fold more aggregates per cell compared to cells expressing no additional tRNA (Figure 6A,B and Figure S6A). The data show that the tRNA^Ser^_AAA_ variant promotes a greater level of FUS-mCherry protein aggregate formation compared to cells expressing no additional tRNA. Although cells with no additional tRNA produced a similar total level of FUS-mCherry protein per cell compared to cells expressing tRNA^Ser^_AAA_ (Figure 5A,C), we observed a significantly greater number of FUS-mCherry aggregates per cell in mistranslating cells compared to cells with high-fidelity translation (Figure 6A,C and Figure S6A). 

### 3.7. FUS R521C Aggregation Kinetics in Normal and Mistranslating Cells

Next, we aimed to quantify the impact of mistranslation on cells expressing a disease-causing FUS allele. Here, we quantified the aggregation kinetics of the ALS-causative FUS R521C allele in cells expressing wild-type or mistranslating tRNA. In cells expressing wild-type tRNA^Ser^_AGA_, FUS R521C-mCherry aggregation kinetics were similar to those we observed for FUS-mCherry, with a 4-fold increase in aggregates per cell compared to cells expressing no additional tRNA (Figure 6C,D and Figures S6B and S8). In cells expressing the tRNA^Ser^_AAA_ mutant and FUS R521C-mCherry, the observed increase in cell death (Figure 4 and Figure S3) contributed to fewer visibly fluorescing cells. 

Repeated cell rupture events in the mistranslating cells lead to saw-tooth-like aggregation kinetics of FUS R521C-mCherry (Supplementary Videos S1 and S2) as dying cells released fluorescent protein aggregates into the medium, causing sudden drops in aggregate counts per cell (Figure 6D and Figure S8). Although cell rupture events occurred in some cells expressing wild-type tRNA, a higher proportion of cells transfected with tRNA^Ser^_AAA_ and FUS R521C ruptured. Indeed, in the middle of the time course, we observed accelerated FUS R521C aggregation in mistranslating cells to the point where, at 51 h post-transfection, mistranslating cells displayed a maximal level of aggregates per cell that was not significantly different from cells expressing wild-type tRNA and FUS R521C (Figure 6D and Figure S6B). The number of FUS R521C aggregates per cell in the tRNA^Ser^_AAA_-expressing cell population reached a plateau level that represents a significant 3-fold increase in aggregates per cell compared to cells expressing no additional tRNA and FUS R521C. At the end of the time course, mistranslating cells and cells expressing wild-type tRNA^Ser^ showed an equivalent level of FUS R521C aggregates per cell that was not significantly different according to statistical analysis (Figure 6D and Figure S6B). The saw-tooth like FUS R521C aggregation kinetics and accompanying cell rupture events help to explain the synthetic toxicity (Figure 4) we observed in mistranslating cells that also express the ALS-causative FUS allele.

### 3.8. Measuring Total FUS Aggregate Levels in Normal and Mistranslating Cells

**Figure 7:**
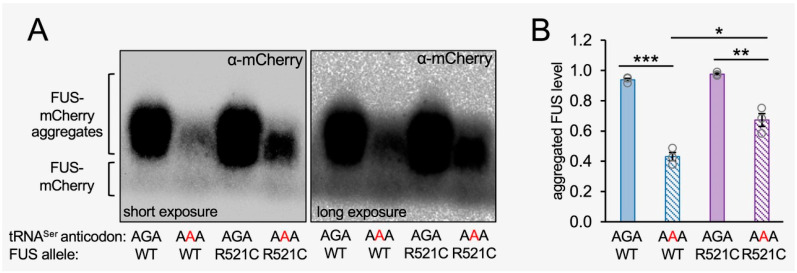
Analysis of total FUS protein aggregate levels in N2a cells expressing wild-type or mistranslating tRNA. Lysates from N2a cells transfected with a plasmid encoding human tRNA^Ser^_AGA_ or tRNA^Ser^_AAA_ and FUS-mCherry or FUS R521C-mCherry were collected at 72 h post-transfection, separated on (**A**) SDD-AGE gels, and Western blotted using an mCherry antibody; short (left panel) and long (right panel) exposure images of the blot are shown. (**B**) The fraction of aggregated FUS protein in the SDD-AGE blots was determined by densitometry. Error bars represent the mean ± 1 standard deviation of at least three biological replicates. Significant differences from pairwise independent sample *t*-tests are indicated (* *p* < 0.05, ** *p* < 0.01, *** *p* < 0.001).

To independently assess the total level of FUS aggregation in each cell line, we measured the fraction of aggregated FUS-mCherry protein in cells expressing tRNA^Ser^_AGA_ or tRNA^Ser^_AAA_ using semi-denaturing detergent agarose gel electrophoresis (SDD-AGE) [41]. SDD-AGE is a semi-quantitative assay that involves partial denaturation (in 0.1% SDS) of aggregated proteins and separation by agarose gel electrophoresis, enabling the resolution of the total level of FUS aggregates in the cell population (Figure 7). A Western blot of the SDD-AGE gel was probed with an mCherry antibody. SDD-AGE separated the lower molecular weight band of monomeric FUS-mCherry from the high-molecular-weight smear corresponding to aggregated FUS-mCherry or FUS R521C-mCherry protein (Figure 7A). The SDD-AGE showed an equivalent level of FUS and FUS R521C aggregates in cells expressing the wild-type tRNA, similar to our observations with fluorescence microscopy (Figure 6 and Figure S6). In agreement with our live cell fluorescence measurements (Figure 5 and Figure 6), the tRNA^Ser^_AAA_ mutant caused a significant reduction in the aggregation of wild-type FUS-mCherry protein aggregates compared to cells expressing wild-type tRNA and wild-type FUS-mCherry. We quantitated a significant 2-fold reduction in the fraction of aggregated wild-type FUS-mCherry in mistranslating cells compared to cells expressing wild-type tRNA^Ser^_AGA_ (Figure 7B).

**Figure 8:**
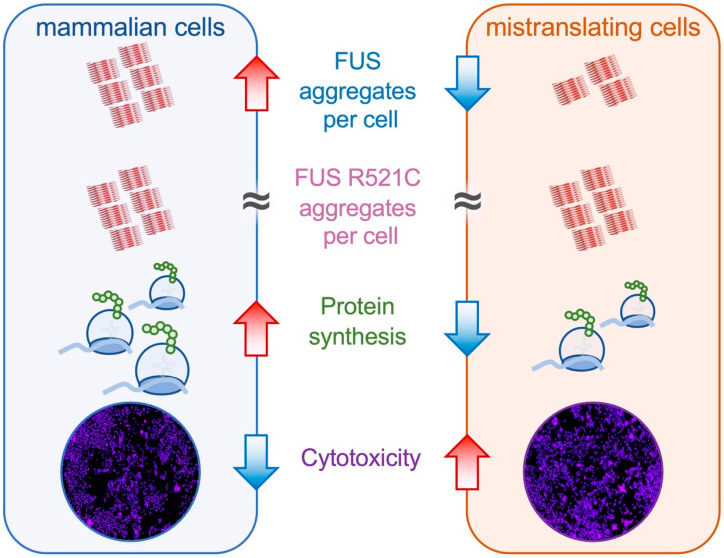
Schematic of FUS aggregation in normal and mistranslating N2a cells. The mistranslating tRNA^Ser^_AAA_ reduces protein levels in cells. We found that FUS aggregation properties and kinetics were characteristically distinct in mistranslating cells. While the wild-type FUS protein showed reduced numbers of FUS aggregates per cell in mistranslating cells, the FUS R521C mutant aggregates were produced at similar numbers per cell in cells expressing wild-type and mistranslating tRNA. We also observed synthetic toxicity in the genetic interaction between the FUS R521C allele and the mistranslating tRNA^Ser^_AAA_ variant.

While our live-cell imaging data indicated a similar number of FUS R521C aggregates per cell in cells expressing wild-type versus mistranslating tRNAs at the end of the time course (Figure 6D and Figure S6B), at 72 h post-transfection in our SDD-AGE experiment, we observed a somewhat reduced level of aggregated FUS R521C in the total population of cells transfected with mutant tRNA that was 75% of the total FUS R521C aggregation level observed in cells expressing wild-type tRNA. Because we observed fewer visibly fluorescing cells expressing mutant tRNA and mutant FUS compared to cells expressing wild-type tRNA and mutant FUS, it is not surprising that the total level of FUS R521C aggregates is somewhat less in mistranslating cells. Still, the observation is in close agreement with the equivalent number of aggregates per cell we identified in individual cells by fluorescent microscopy (Figure 6D). In further agreement with our single-cell observations (Figure 6 and Figure S6), we also observed significantly more FUS R521C aggregation compared to wild-type FUS aggregation in mistranslating cells expressing tRNA^Ser^_AAA_ (Figure 7B). The data confirm our observations of the increased aggregate formation of the ALS-causative FUS variant relative to the wild-type FUS allele in mistranslating cells (Figure 8).

## 4. Discussion

### 4.1. De-Regulated Protein Homeostasis in ALS and in Cells Expressing Mutant or Wild-Type tRNAs

Studies in N2a cells [48], HEK 293T cells [52], and mice [53] have each documented that expression of mutant human FUS proteins causes down-regulation of protein synthesis. Compared with wild-type FUS, N2a cells expressing ALS-linked mutant R495X and P525L caused a 20–30% reduction in protein production levels generally, but the level of FUS and mutant FUS produced was similar [48]. FUS aggregation can inhibit protein synthesis through multiple mechanisms that include the disruption of nonsense-mediated decay [48], modulation of cellular signaling via the mechanistic target of rapamycin complex (mTORC) [52], or induction of the integrated stress response [53] pathways. FUS mutations also indirectly affect protein synthesis by causing complex dysregulation of transcription [54] and RNA metabolism [55]. We and others have shown that amino acid mis-incorporation resulting from mutations in tRNA genes [17,42,56,57], including the tRNA^Ser^_AAA_ variant we investigated here, or aminoacyl-tRNA synthetase mutants [58] can likewise down-regulate protein synthesis. The fact that mistranslation and FUS protein aggregation both disrupt protein homeostasis in cells helps to explain the synthetic toxicity of their genetic interaction.

In mistranslating cells, the observed synthetic toxicity with FUS R521C resulted from changes in FUS aggregate formation rather than in total FUS protein levels. For both FUS alleles, compared to cells with no additional tRNA, we observed significantly more FUS aggregates per cell in mistranslating cells despite comparable FUS protein levels, demonstrating a dominant negative effect on FUS aggregation in mistranslating cells. A key finding of our paper is that the FUS R521C aggregation kinetics in mistranslating cells are distinct from both FUS R521C aggregation in N2a cells expressing wild-type tRNA and in mistranslating cells expressing the wild-type FUS. In agreement with previous observations [59], we observed little difference between FUS and FUS R521C aggregation in wild-type cells; however, differences in FUS and FUS R521C aggregation emerged in mistranslating cells.

Indeed, the genetic interaction of mistranslation and FUS protein aggregation was particularly remarkable in our observations of FUS R521C aggregation kinetics in mistranslating cells (Figure 8). Co-expression of the tRNA^Ser^_AAA_ variant and FUS R521C had a potent synthetic toxic effect, as evidenced in a quantitative cell death assay. Despite reduced FUS R521C production in cells expressing the mutant versus the wild-type tRNA^Ser^, we observed a similar plateau level in the number of FUS R521C aggregates per cell in mistranslating and normal cells. Finally, we documented rapid FUS R521C aggregation followed by cell rupture events in mistranslating cells, further demonstrating the synthetic toxicity of mistranslation with FUS R521C. The mistranslating cells expressing FUS R521C were coping with two simultaneous stressors on protein homeostasis: mistranslation and protein aggregation. The mistranslating cells died at a higher rate because the cell’s ability to simultaneously manage both mis-made proteins and aggregating R521C protein was exceeded. Other cell stressors, such as oxidative stress and heat stress, enhanced the aggregation of other ALS-linked FUS mutants, including R495X, H517Q, and R521G [60]. 

### 4.2. Considerations of Transfer RNA Variants as Therapeutics

The fact that our data show that wild-type tRNA^Ser^ gene increased the protein production and aggregation of FUS and FUS R521C has interesting implications for identifying applications and pitfalls of tRNAs as therapeutic molecules. The upregulation of tRNA expression is generally recognized to enhance metabolic activity and cell proliferation [61,62], and increased tRNA levels promote protein synthesis in human cancer cells [63]. Since tRNA overexpression increases protein production, it could be applied in cells where protein synthesis is chronically repressed, a hallmark of ALS [48,52,53] and other neurodegenerative diseases such as Huntington’s disease [64]. In the context of FUS, however, our data show that wild-type tRNA^Ser^ overexpression increases protein aggregation associated with ALS.

Nevertheless, the use of either cognate tRNA supplementation or the application of stop codon suppressor tRNAs is driving the rapidly emerging area of tRNA medicine [65]. Indeed, therapeutic tRNA overexpression can rescue peripheral neuropathy in animal and cellular models of Charcot–Marie–Tooth disease caused by mutations in glycyl-tRNA synthetase, demonstrating the potential of tRNA therapeutics [66]. CMT and other disease-causing mutations in several different tRNA synthetases, such as histidyl-tRNA synthetase [67], may also be amenable to treatment with cognate tRNAs. A different approach demonstrated the use of nonsense suppressor tRNAs to cure mucopolysaccharidosis type I, a disease caused by a premature stop codon [68]. As drugs, tRNAs are well tolerated in cells [69,70] and mice [68,69], effective in correcting genetic defects, and easily delivered to cells or mice as genes or as synthetic RNAs by plasmid transfection [17], adeno-associated viral vectors (AAVs) [68], or lipid nanoparticles (LNPs) [69].

### 4.3. Transfer RNA Variants as Modifiers of Neurodegenerative Diseases

Considering the diversity of human tRNA gene variants in the population that could cause errors in protein synthesis [6,14,19,21], our data presented here add strong support to our hypothesis that although naturally occurring tRNA variants are tolerated under normal conditions [14], tRNA-dependent mistranslation may exacerbate the toxicity associated with protein misfolding and aggregation in neurodegenerative diseases [6,17]. Certainly, tRNA mutations only represent one route to generate mistranslation in cells that may cause or worsen a disease. Indeed, mistranslation and disease can be caused by defects in the AARS editing of mis-charged tRNAs. For example, the alanyl-tRNA synthetase (AlaRS) Ala734Glu variant is unable to hydrolyze mis-aminoacylated tRNA^Ala^ causing Ser mis-incorporation at Ala codons. The AlaRS variant causes a sticky fur phenotype in mice and an accompanying loss of cerebellar Purkinje cells [71] through increased apoptosis. 

Both loss-of-function and gain-of-function tRNA variants have the strong potential to modify or exacerbate neurodegenerative diseases. While mutations in AARSs and other protein synthesis components, including elongation factors and tRNA-modifying enzymes, have also been linked to neurodegenerative disease [7,72], research on tRNA variants in neurodegenerative disease is an open area for new investigations. Because there are many copies of most human tRNA genes, ongoing work is seeking to understand how mutations in single tRNAs can also cause or contribute to disease [3,6,14,17]. Unlike the AlaRS example above that causes some fraction of mis-aminoacylation on all tRNA^Ala^ isoacceptors, a single tRNA mutant must compete with and may be compensated for by other tRNA gene copies that read the same codon or codons. 

Recent work has identified links between a loss-of-function tRNA mutation and neurodegenerative phenotypes in mice [57]. Mice harboring a single mutant tRNA^Arg^_UCU_ C50T had defects in synaptic transmission in neuronal cells and increased seizure susceptibility [73]. In the same study, partial CRISPR genetic knockouts in mouse forebrains demonstrated that the deletion of another tRNA gene (tRNA-Ile-TAT-2-3) stimulated the integrated stress response [73]. Our own work on cellular models of Huntington’s disease [17] found reduced huntingtin expression and slowed aggregation kinetics of disease-linked huntingtin proteins in cells expressing tRNA^Ser^_AAA_. Mistranslating cells also displayed defective polyQ aggregate degradation; hence, our findings suggested that the tRNA mutant has the potential to alter the age of onset and increase the severity of Huntington’s disease. In the case of ALS, mistranslation greatly modified the kinetics of FUS aggregation and enhanced toxicity associated with an ALS-causative allele. In future work, beyond the scope of this study, we will investigate the genetic interaction of natural human tRNA mutants with other ALS-causative alleles.

ALS affects 5 in every 100,000 individuals worldwide [74]. Approximately 10% of ALS cases are familial or inherited forms of the disease caused by a dominant mutation in FUS or several other causative alleles. The severity and age of onset of ALS can vary greatly even among individuals with the same causative mutant [32]. For example, in ALS patients with FUS mutations, the mean age of onset is 41.8 years, with a standard deviation of 14.5 years [75]. Patients with FUS R521 mutations show a median age of onset of just more than 40 years, with a range of 20 to 80 years in the presentation of the disease [76]. Thus, there is a need to identify additional genetic modifiers of ALS that impact disease progression.

Our data suggest that mistranslating tRNA mutants may act as genetic modifiers of FUS protein aggregation. Moreover, tRNA^Ser^_AAA_ is just one example of a mistranslating tRNA that we identified in human genomes [6,21]. Humans encode both more common as well as rare tRNA variants that are likely to cause mistranslation in cells [6,14,17,21]. The tRNA^Ser^ G35A allele is found in ~2% of individuals in our general population [6]. The frequency for this allele (rs147439337) is consistent across large genome databases, including in the 1000 Genomes Project (1.8% allele frequency) [6,19,77], the GnomAD database of >140,000 human genomes (1.76% allele frequency) [78], and our own targeted sequencing studies of all human tRNA genes (3% allele frequency) [21]. Based on this allele frequency, we anticipate that thousands of ALS patients may also have this mistranslating tRNA mutant in their genetic background. The MINE project annotated variants in protein-coding genes from >1500 ALS patient whole-genome sequences, which will be an invaluable resource to identify tRNA variants in ALS patients once information on tRNA gene variants is made available [79]. Analyses of these data and our future experimental work will identify additional human tRNA variants that cause mistranslation and the consequences of genetic interactions between mistranslating tRNAs and alleles that cause neurodegeneration. 

## Data Availability

All data are available in the Figures and Supplementary Material.

## Supplementary Materials

The following supporting information can be downloaded at: https://www.mdpi.com/article/10.3390/genes14020518/s1, Supplementary Methods; Supplementary References [80,81,82,83,84,85]; Figure S1: Images of N2a cells transfected with wild-type or mistranslating tRNAs prepared for tRNA sequencing; Figure S2: Volcano plot of tRNA abundance in wild-type and mistranslating cells; Figure S3: Cytotoxicity in N2a cells co-expressing tRNA^Ser^ variants with mCherry or FUS-mCherry variants; Figure S4: Total FUS-mCherry levels in N2a cells expressing wild-type or mistranslating tRNA; Figure S5: FUS aggregate formation in cells expressing wild-type tRNA^Pro^ or the alanine-accepting tRNA^Pro^ G3:U70 variant; Figure S6: Quantitation of FUS aggregate levels in N2a cells expressing wild-type or mistranslating tRNA; Figure S7: Additional images of FUS-mCherry aggregates in N2a cells; Figure S8: Additional images of FUS R521C-mCherry aggregates in N2a cells; Table S1: Oligonucleotide sequences; Supplementary Video S1: The supplementary video file shows representative fluorescence microscopy images of the time course of FUS aggregation kinetics shown in Figure 5; Supplementary Video S2: The supplementary video file shows additional representative fluorescence microscopy images of the time course of FUS R521C aggregation kinetics shown in Figure 5; Supplementary Data File S1: Excel spreadsheet containing raw read counts for tRNA sequencing of N2a cells expressing tRNA^Ser^_AGA_ or tRNA^Ser^_AAA_; Supplementary Appendix: Image J macros for analysis of FUS-mCherry fluorescence and aggregates per cell.
