# Supplementary material for: Genetic Interaction of tRNA-Dependent Mistranslation with Fused in Sarcoma Protein Aggregates
Source: Genes (Basel). 2023 Feb 18;14(2):518. doi: 10.3390/genes14020518 (PMC9956149; doi:10.3390/genes14020518)
Supplement: Supplementary file 1 [file genes-14-00518-s001.zip › Lant-etal-2023_SI/Lant-etal-2023_SI.pdf]

Supplementary Material for:

Genetic interaction of tRNA-dependent mistranslation with fused in sarcoma protein aggregates

Jeremy T. Lant[1], Farah Hasan[1], Julia Briggs[1], Ilka U. Heinemann[1] & Patrick O'Donoghue [1,2,*]

[1] Department of Biochemistry, The University of Western Ontario, London, Ontario N6A 5C1, Canada.
[2] Department of Chemistry, The University of Western Ontario, London, Ontario N6A 5C1, Canada.
* Correspondence: patrick.odonoghue@uwo.ca

S.1 SUPPLEMENTARY METHODS

Cell harvesting and western blotting. At 48 or 72 hours post-transfection, as indicated, cells grown in 6-well plates were lifted in phosphate buffered saline (PBS; 137 mM NaCl, 2.7 mM KCl, 10 mM Na_2HPO_4, 1.8 mM KH_2PO_4, pH 7.4) with 10 mM EDTA for 10 min at 37 °C, collected in sterile 1.5 mL microcentrifuge tubes, and pelleted by centrifugation at 300 × g for 3 min at 4 °C. The supernatant was aspirated, and cell pellets were stored at -80 °C. Cell pellets were resuspended in 90 µL of mammalian cell lysis buffer containing 50 mM Na_2HPO_4, 1 mM $Na_4P_2O_7$, 20 mM NaF, 2 mM EDTA, 2 mM EGTA, 1 mM Triton X-100, 1 mM dithiothrieitol, 0.3 mM phenylmethylsulfonyl fluoride, and 1 tablet/10 ml complete mini EDTA-free Protease Inhibitor Cocktail (Roche, Mississauga, ON), incubated for 5 min on ice, and centrifuged at 21,000 × g for 10 min at 4 °C. Supernatants were collected in 1.5 mL microcentrifuge tubes and kept on ice or stored at -20 °C for up to one week.

Protein concentrations in the lysates were determined with a Pierce bicinchoninic acid (BCA) protein assay kit (ThermoFisher). Lysates were diluted to equal concentrations with sterile double distilled H_2O and 3 × sodium dodecyl sulfate (SDS) loading dye (0.5 M Tris-HCl, pH 6.8; 1.12 M sucrose; 0.025% w/v bromophenol blue; 3.8% w/v SDS). Lysate corresponding to 12 µg total soluble protein was separated by SDS-polyacrylamide gel electrophoresis (10% acrylamide) with protein standards (BioRad, Hercules, CA, USA) for size determination. Proteins were transferred to methanol-activated polyvinylidene fluoride membranes using a Trans-Blot Turbo Transfer System (25 V, 1.3 A for 14 min; BioRad). Blocking and washing solutions were prepared in tris-buffered saline (TBS; 50 mM Tris-HCl, pH 7.5, 150 mM NaCl). Membranes were incubated for 1 hour in blocking solution (3% bovine serum albumin (BSA), 0.1% Tween 20, 1X TBS) before adding primary antibodies at a 1:1000 (α-mCherry, Abcam, ab213511) or 1:5000 (α-GAPDH, Sigma-Aldrich, MAB374m) dilution in blocking solution. Membranes were incubated with primary antibody in blocking solution overnight at 4 °C, washed 3 × 10 min in washing solution (1% BSA, 0.1% Tween 20, 1X TBS), then incubated with anti-mouse (ThermoFisher, MA1-21315) or anti-rabbit (Sigma-Aldrich, GENA9340) horse radish peroxidase-linked secondary antibodies for 2 hours at room temperature with a 1:2000 final dilution. Membranes were washed with 1 × TBS with 0.1% Tween 20 for 3 × 10 min, followed by one wash for 10 minutes in 1 × TBS. Proteins were visualized using Clarity Western enhanced chemiluminescence (ECL) Substrates (Bio-Rad) following the manufacturer's instructions and imaged with a ChemiDoc MP System (Bio-Rad).

tRNA extraction and demethylation. Cells were harvested and stored in TRIzol reagent in liquid nitrogen and sent to Arraystar, Inc (Rockville, MD, USA) for tRNA sequencing. Total RNA was isolated using Trizol reagent (Invitrogen), and the quality of total RNA samples was checked using denaturing agarose electrophoresis, and 28S and 18S ribosomal bands were visually inspected. Total RNA concentration was quantified measuring absorbance at 260 nm on a NanoDrop ND-1000. tRNA was isolated from total RNA using Denaturing Urea Polyacrylamide Gel Electrophoresis (Urea PAGE). 2μg total RNA per sample was resolved on 7.5% Urea PAGE gels (7M Urea) and small RNAs recovered within a size window of 60-100 nt for tRNA. tRNA demethylation was carried out using rtStar tRF&tiRNA Pretreatment Kit (Arraystar, AS-FS-005) for tRNA m1A and m3C demethylation. Briefly, 50 μL demethylation reaction mixture was prepared according to the manufacturer's protocol and incubated at 37 °C for 2 hr. Then 40 μL nuclease-free water and 10 μL 5× Stop Buffer were added to terminate the reaction. Demethylated tRNA was purified by phenol-chloroform extraction and ethanol precipitation. Demethylated tRNA was partially hydrolyzed according to Hydro-tRNAseq method with some modifications [80]. tRNA was subjected to limited alkaline hydrolysis in 15 μL hydrolysis buffer (10 mM Na_2CO_3, 10mM $NaHCO_3$, pH 9.7) at 90 °C for 7 min. The partially hydrolyzed tRNA was dephosphorylated with 10 U Calf Intestinal Alkaline Phosphatase (CIP; New England Biolabs, M0290L) in a 50 μL reaction of CIP buffer (100mM NaCl, 50 mM Tris-HCl, pH 7.9, 10 mM $MgCl_2$, 1mM DTT, 3mM Na_2CO_3, 3mM $NaHCO_3$) at 37 °C for 1 hr. The resulting tRNA was purified with TRIzol reagent and then re-phosphorylated with 10 U T4 polynucleotide kinase (New England Biolabs, M0201L) in a 20 μL reaction in T4 buffer (70 mM Tris-HCl, pH 7.6, 10 mM $MgCl_2$, 5 mM DTT, 1 mM ATP) at 37 °C for 1 hour, and again purified with TRIzol reagent.

Library preparation and tRNA sequencing. Partially hydrolyzed tRNA fragments were converted to barcoded small RNA sequencing libraries using NEBNext Multiplex Small RNA Library Prep Set for Illumina kit (New England Biolabs, E7300L/E7850L) according to manufacturer's instructions. The procedures generally included: 1) 3′-adapter ligation; 2) 3′-adapter blocking; 3) 5′-adapter ligation; 4) reverse transcription; 5) PCR amplification; 6) size selection of ~140-155 bp PCR amplified fragments (corresponding to ~19-35nt tRNA fragments) using 6% PAGE. The completed libraries were qualified and quantified by Agilent 2100 Bioanalyzer. Equal amounts of Hydro-tRNAseq libraries were mixed and denatured with 0.1 M NaOH to generate single-stranded DNA molecules, and loaded onto the reagent cartridge (NextSeq 500/550 High-Output v2 kit). Sequencing was performed on Illumina NextSeq 500 system with sequencing 50 cycles. Sequencing quality was examined by FastQC software[1] and trimmed reads (pass Illumina quality filter, trimmed 3′-adaptor bases by cutadapt [81]) were aligned to the cytoplasmic mature-tRNA sequence getting from GtRNAdb [20] and the mitochondrial mature-tRNA sequences getting from mitotRNAdb [82] using BWA [83] software. For tRNA alignment, the maximum mismatch was 2 [80]. The tRNAs expression profiling was calculated based on uniquely mapped reads and including mapped reads, respectively. The differentially expressed tRNAs were screened based on the count value with R package edgeR [84].

Cytotoxicity assay. Cytotoxicity was determined using a dye-exclusion assay with SYTOX Blue Dead Cell Stain (Invitrogen). After 48 hours, cells were washed once with Hank's buffered salt solution (HBSS) and the media was replaced with DMEM containing 1 μM SYTOX Blue Dead Cell Stain. SYTOX Blue fluorescence intensity was measured with a Synergy H1 plate reader with a monochromatic filter set to 444 nm excitation and 480 nm emission. Images were captured using an EVOS FL auto fluorescent

[1] http://www.bioinformatics.babraham.ac.uk/projects/fastqc/

microscope using an RFP filter cube (531 ± 40 nm excitation, 593 ± 40 nm emission) to measure mCherry fluorescence and CFP filter cube (445 ± 45 nm excitation, 510 ± 42 nm emission) to measure SYTOX Blue fluorescence. To establish the total number of cells, 0.25% v/v Triton X-100 was added to each well and the cells were incubated at 37 °C for 40 min to completely permeate cell membranes and allow SYTOX Blue staining. Images were captured again from the same location using the EVOS microscope and SYTOX Blue fluorescence was measured again with the Synergy H1 after Triton X-100 treatment. Cell death levels were calculated as the ratio SYTOX blue fluorescence before and after Triton X-100 treatment.

Statistical analysis. Statistical analyses used in each figure are referenced in the figure legends. Independent sample t-tests were done by pairwise comparison of groups of biological means as indicated.

S.2 SUPPLEMENTARY REFERENCES (see main text for full reference list)

3. Lant, J.T.; Berg, M.D.; Sze, D.H.W.; Hoffman, K.S.; Akinpelu, I.C.; Turk, M.A.; Heinemann, I.U.; Duennwald, M.L.; Brandl, C.J.; O'Donoghue, P. Visualizing tRNA-dependent mistranslation in human cells. *RNA Biol* **2018**, *15*, 567-575.
17. Lant, J.T.; Kiri, R.; Duennwald, M.L.; O'Donoghue, P. Formation and persistence of polyglutamine aggregates in mistranslating cells. *Nucleic Acids Res* **2021**, *49*, 11883-11899.
20. Chan, P.P.; Lowe, T.M. GtRNAdb 2.0: an expanded database of transfer RNA genes identified in complete and draft genomes. *Nucleic Acids Res* **2016**, *44*, D184-189.
49. Klickstein, J.A.; Mukkavalli, S.; Raman, M. AggreCount: an unbiased image analysis tool for identifying and quantifying cellular aggregates in a spatially defined manner. *J Biol Chem* **2020**, *295*, 17672-17683.
80. Gogakos, T.; Brown, M.; Garzia, A.; Meyer, C.; Hafner, M.; Tuschl, T. Characterizing Expression and Processing of Precursor and Mature Human tRNAs by Hydro-tRNAseq and PAR-CLIP. *Cell Rep* **2017**, *20*, 1463-1475.
81. Martin, M. Cutadapt remoes adapter sequences from high-throughput sequencing reads. *EMBnet.journal* **2011**, 17, 1, 10-12.
82. Juhling, F.; Morl, M.; Hartmann, R.K.; Sprinzl, M.; Stadler, P.F.; Putz, J. tRNAdb 2009: compilation of tRNA sequences and tRNA genes. *Nucleic Acids Res* **2009**, *37*, D159-162.
83. Li, H.; Durbin, R. Fast and accurate short read alignment with Burrows-Wheeler transform. *Bioinformatics* **2009**, *25*, 1754-1760.
84. Robinson, M.D.; McCarthy, D.J.; Smyth, G.K. edgeR: a Bioconductor package for differential expression analysis of digital gene expression data. *Bioinformatics* **2010**, *26*, 139-140.
85. Schneider, C.A.; Rasband, W.S.; Eliceiri, K.W. NIH Image to ImageJ: 25 years of image analysis. *Nat Methods* **2012**, *9*, 671-675.

S.3 SUPPLEMENTARY TABLE

Table S1. Oligonucleotide sequences

Description	Nucleotide sequence (5′-3′)
FUS cloning in pcDNA3.1 NheI/HindIII sites (Fwd)	CAGACTGCTAGCATGGCCTCAAACGATTATACCC
FUS cloning in pcDNA3.1 NheI/HindIII sites (Rev with linker sequence)	CAGACTAAGCTTGCCTCCAGACCCTCCGCCATACGGCCTCTCCCTGC
FUS R521C round-the-horn mutagenesis (Fwd)	TGCAGGGAGAGGCCGT
FUS R521C round-the-horn mutagenesis (Rev)	ATCCTGTCTGTGCTCACCC
FUS cloning in WT-Pan NheI/SpeI sites (Fwd)	CAGACTGCTAGCATGGCCTCAAACGATTATACCC
FUS cloning in WT-Pan NheI/SpeI sites (Rev with linker sequence)	CAGACTACTAGTGAGCCTCCAGACCCTCCGCCATACGGCCTCTCCCTGC

S.4 SUPPLEMENTARY FIGURES

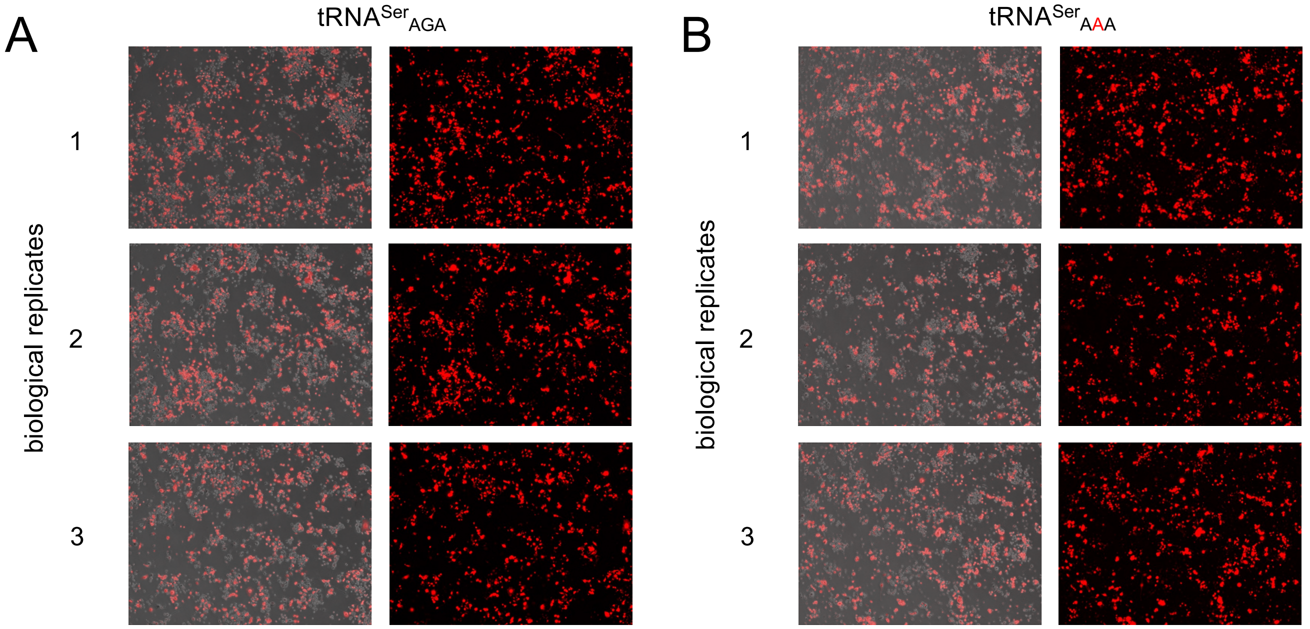

Figure S1. Images of N2a cells transfected with wild-type or mistranslating tRNAs prepared for tRNA sequencing. N2a cells were transfected with a plasmid encoding human (A) wild-type $tRNA^{Ser}_{AGA}$ or (B) $tRNA^{Ser}_{AAA}$ and mCherry. Representative brightfield overlays (left) with fluorescent microscopy images (RFP, ex. 531 nm, em. 593 nm, right) were used to estimated transfection efficiency.

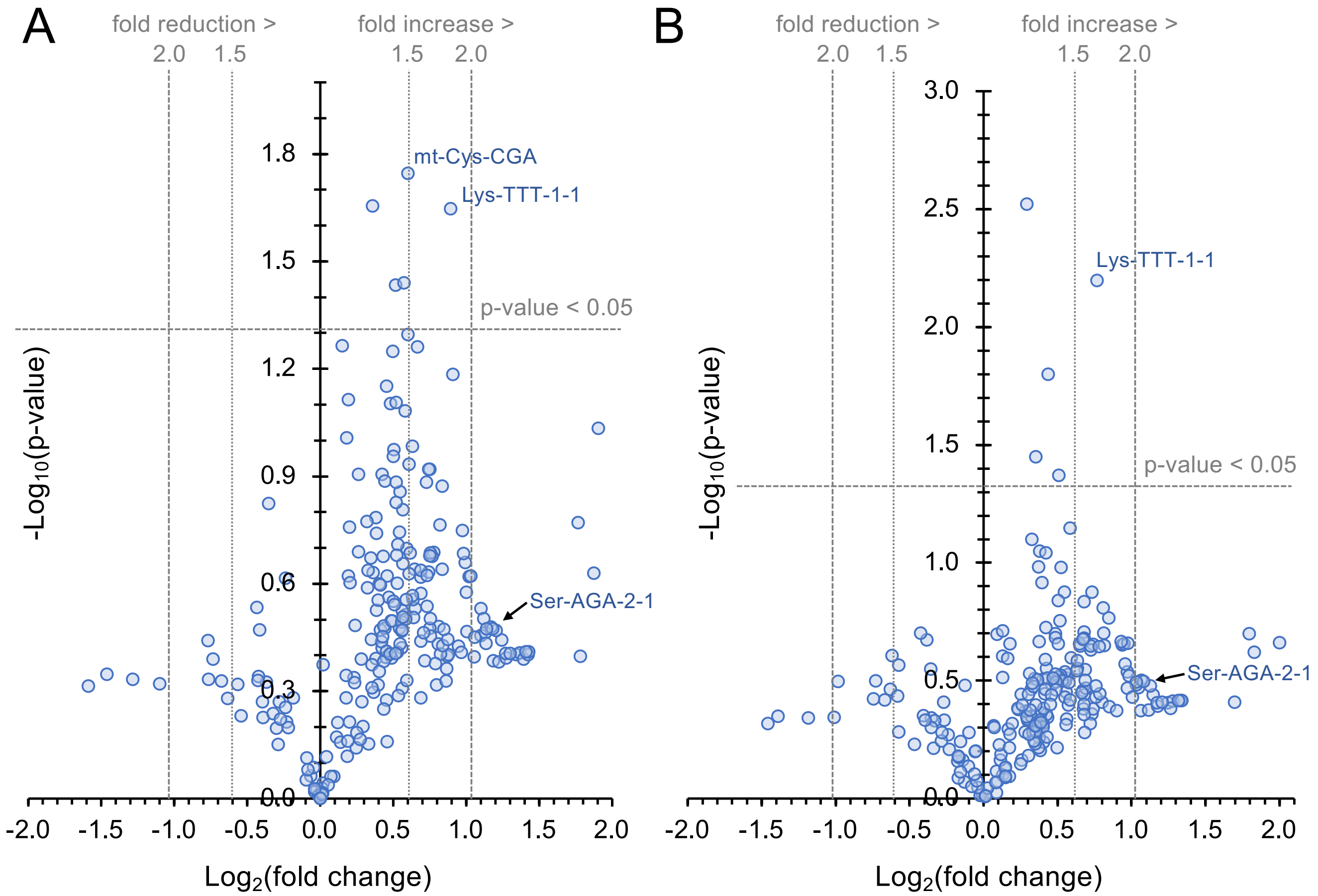

Figure S2. Volcano plot of tRNA abundance in wild-type and mistranslating cells. tRNA expression was plotted as Log_2(fold change) in read counts from N2a cells expressing wild-type $tRNA^{Ser}{}_{AGA}$ divided by read counts from N2a cells expressing mutant $tRNA^{Ser}{}_{AAA}$ versus the significance, $-Log_{10}$(p-value). The tRNA sequencing data is plotted according to (A) raw read counts and to (B) read counts normalized by the total number of reads from each biological replicate. Positive Log_2(fold change) indicates tRNAs relatively up-regulated in the wild-type cell line, while negative Log_2(fold change) values represent tRNAs relatively up-regulated in cell lines expressing the mistranslating tRNA. No tRNA transcripts (blue dots) were significantly and ≥ 2-fold changed in abundance. According to (A) raw read counts two tRNA genes (Lys-TTT-1-1, mitochondrial Cys-CGA) or (B) according to normalized read counts one tRNA gene (Lys-TTT-1-1) showed more than a 1.5-fold significant increase in abundance (p-value ≤ 0.05, fold-change ≥ 1.5, fold-change ≤ 2) in cells expressing the wild-type $tRNA^{Ser}$. The Ser-AGA-2-1 tRNA pool was not significantly changed.

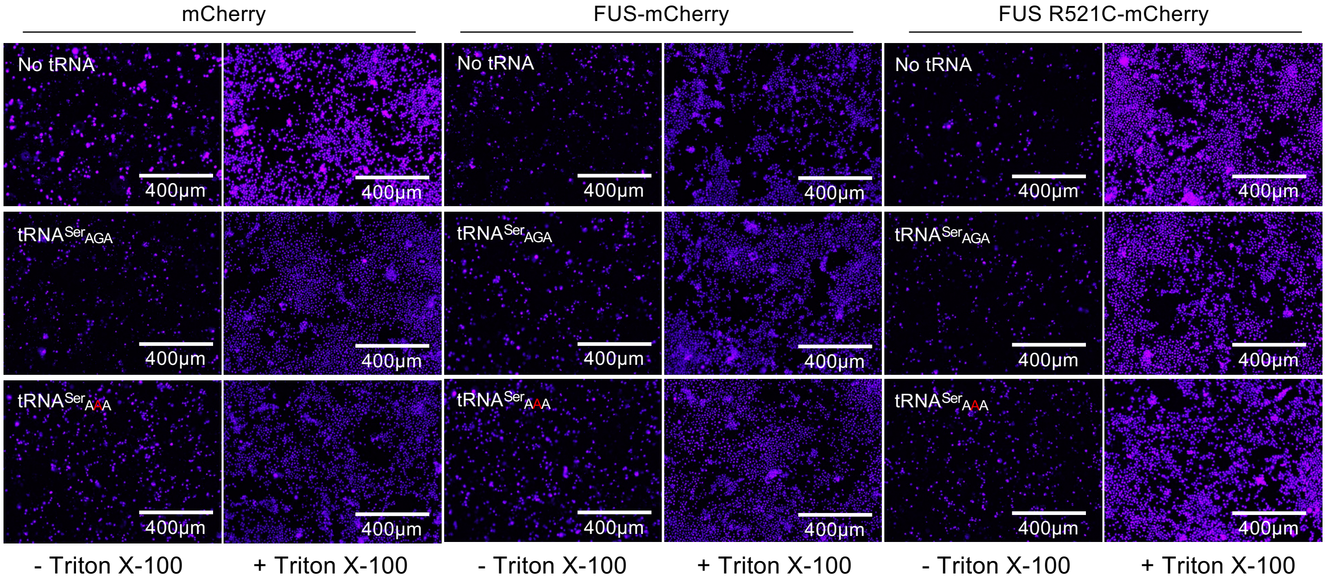

Figure S3. Cytotoxicity in N2a cells co-expressing tRNA^{Ser} variants with mCherry or FUS-mCherry variants. N2a cells were transfected with a plasmid encoding no tRNA, human tRNA$^{Ser}_{AGA}$, or tRNA$^{Ser}_{AAA}$ along with mCherry, human FUS-mCherry, or FUS R521C-mCherry. After 72-hours post-transfection, cytotoxicity was assayed with a dye exclusion assay using Sytox blue nucleic acid stain. The images are representative of images from the five biological replicates included in Fig. 4. The images were captured by fluorescence microscopy (CFP; ex. 445 nm, em. 510 nm).

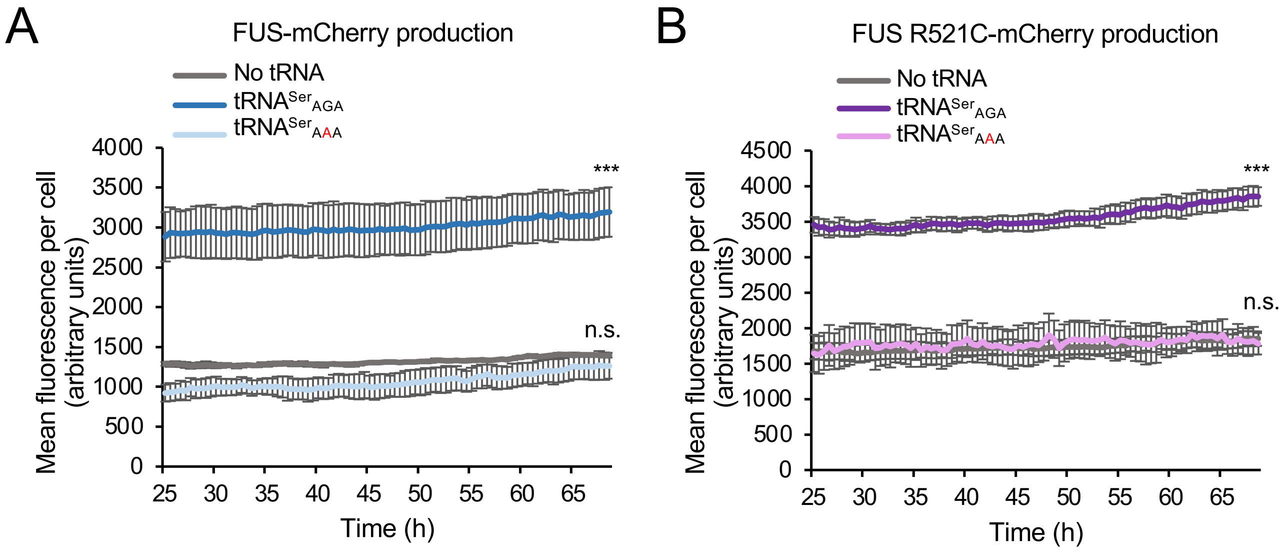

Figure S4. Total FUS-mCherry levels in N2a cells expressing wild-type or mistranslating tRNA. N2a cells were transfected with a plasmid encoding no tRNA, human $tRNA^{Ser}_{AGA}$ or G35A variant $tRNA^{Ser}_{AAA}$ and human FUS-mCherry or FUS R521C-mCherry. Images (see Fig. 5) of fluorescing cells were captured by live-cell fluorescence microscopy (RFP, ex 531 nm, em 593 nm) beginning 24 hours after transfection for a 43.5-hour time lapse. Total (A) FUS-mCherry and (B) FUS R521C-mCherry levels are represented by the mean mCherry fluorescence observed every 30 minutes during the time course shown in Fig. 5. Total mCherry fluorescence per cell was quantitated with a custom Fiji/ImageJ macro (see Supplementary Appendix). Error bars represent the mean ± 1 standard deviation of at least five biological replicates. Significant differences from pairwise independent sample t-tests are indicated (n.s. – not significant, *** $p < 0.001$).

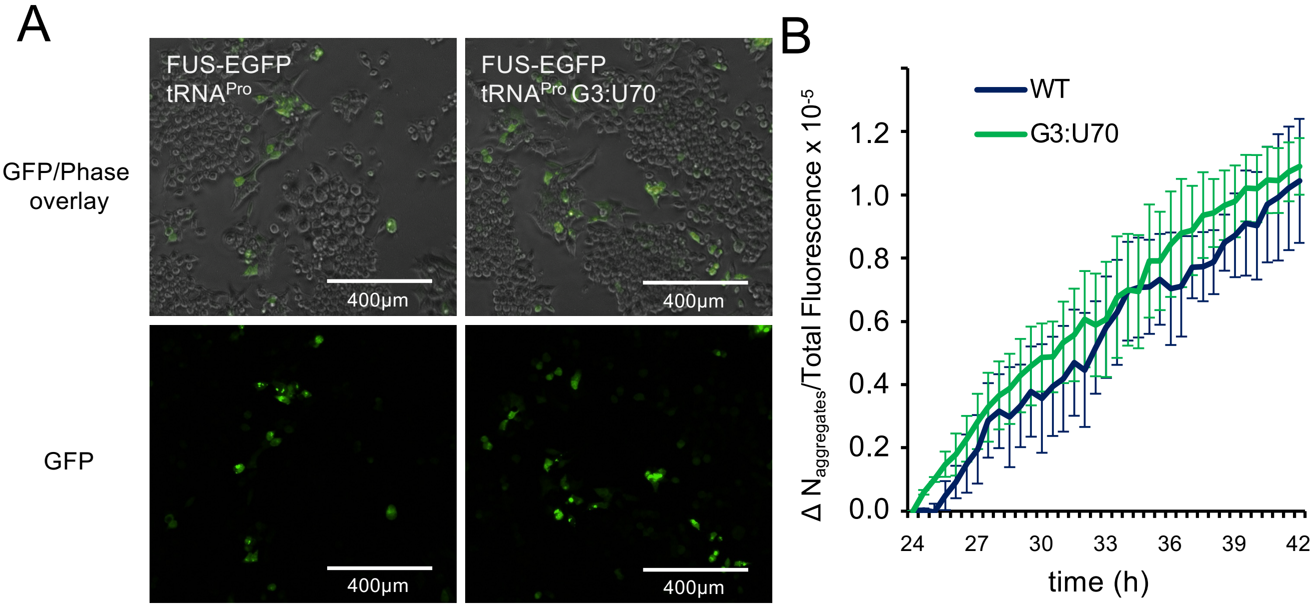

Figure S5. FUS aggregate formation in N2a cells expressing wild-type tRNA^{Pro} or the alanine accepting tRNA^{Pro} G3:U70 variant. N2a cells were transfected with a plasmid encoding human FUS-EGFP fusion protein and wild-type human tRNA^{Pro} or a tRNA^{Pro} G3:U70 variant that decodes proline codons with alanine [3]. (A) Representative images were captured by fluorescence microscopy (GFP; ex 470 nm, em 510 nm) (bottom) and overlaid with bright field images of the cells (top). (B) Starting 24 hours pos-transfection, the change in the number of aggregates over total cell fluorescence was quantified over an 18-hour live-cell imaging time-course with a custom Fiji/ImageJ macro (see Supplementary Appendix) as previously described [17]. Error bars represent the mean ± 1 standard deviation of at least three biological replicates.

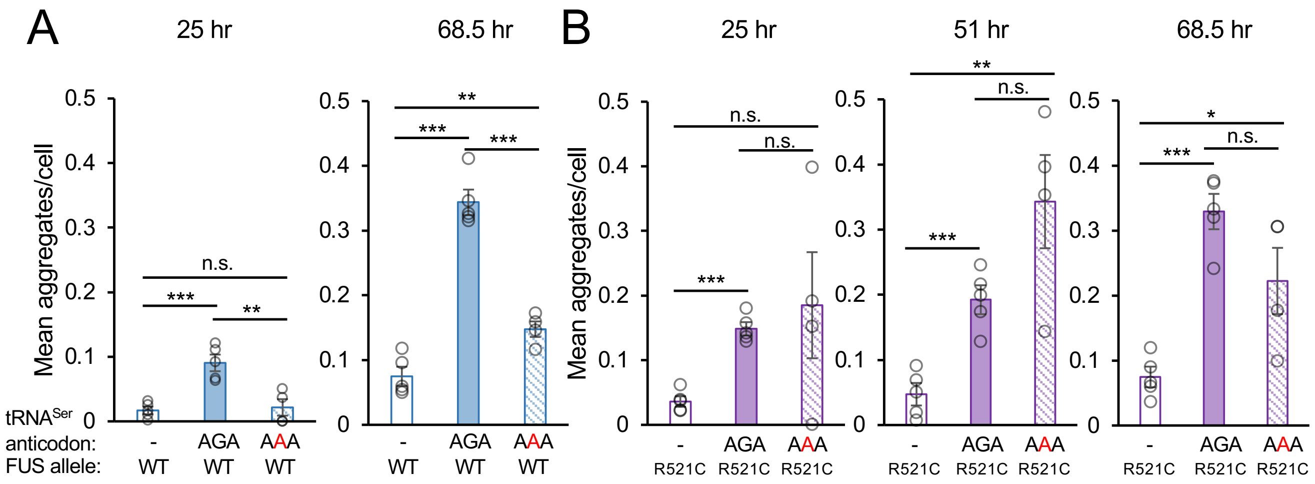

Figure S6. Quantitation of the mean number of FUS aggregates per cell in N2a cells expressing wild-type or mistranslating tRNA. N2a cells were transfected with a plasmid encoding no tRNA, human $tRNA^{Ser}_{AGA}$, or $tRNA^{Ser}_{AAA}$ and (A) FUS-mCherry or (B) FUS R521C-mCherry. The data are based on analysis of the images shown in Figs. 6, S7, and S8. The mean number of (A) FUS-mCherry or (B) FUS R521C-mCherry aggregates per cell was plotted at the indicated timepoints. Error bars represent the mean number of aggregates per cell ± 1 standard deviation of at least four biological replicates. Significant differences from pairwise independent sample t-tests are indicated (n.s. – not significant, * $p < 0.05$, ** $p < 0.01$, *** $p < 0.001$).

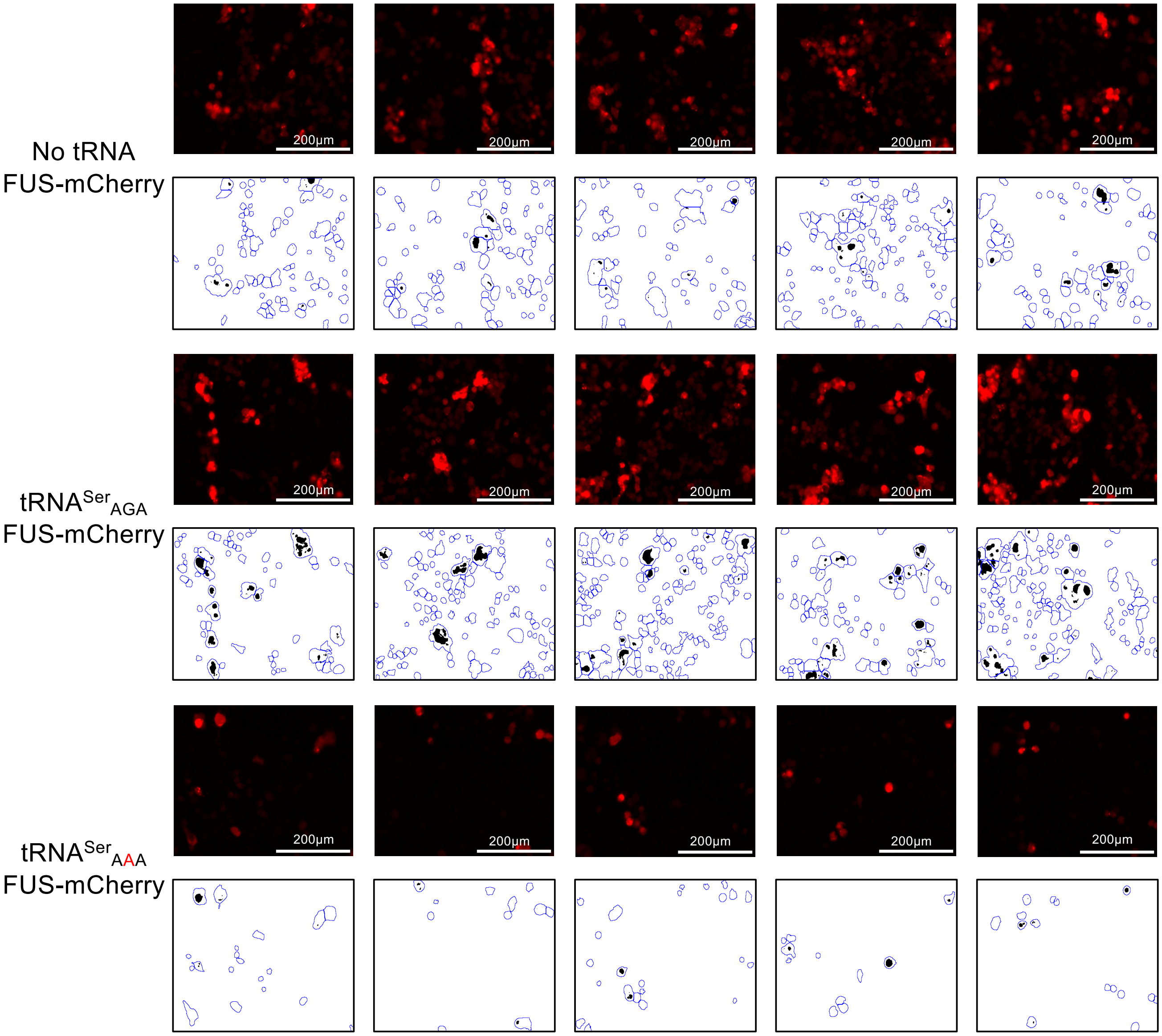

Figure S7. Additional images of FUS-mCherry aggregates in N2a cells. N2a cells were transfected with a plasmid encoding no tRNA (top), human tRNA$^{Ser}_{AGA}$ (middle), or tRNA$^{Ser}_{AAA}$ (bottom) and FUS-mCherry**.** Representative images (see also Figs. 6) of fluorescing cells were captured by live-cell fluorescence microscopy (RFP ex. 531 nm, em. 593 nm) beginning 25 hours after transfection for a 43.5-hour time course. Fluorescent cell area representing cells and aggregates was determined using a custom Fiji/ImageJ macro. Cell and aggregate counts were determined based on the observed median cells and aggregate area (see Supplementary Appendix). Representative images of the fluorescing cells (above) and black and white mask images (below) showing aggregate area (black) and cell area (blue outlines).

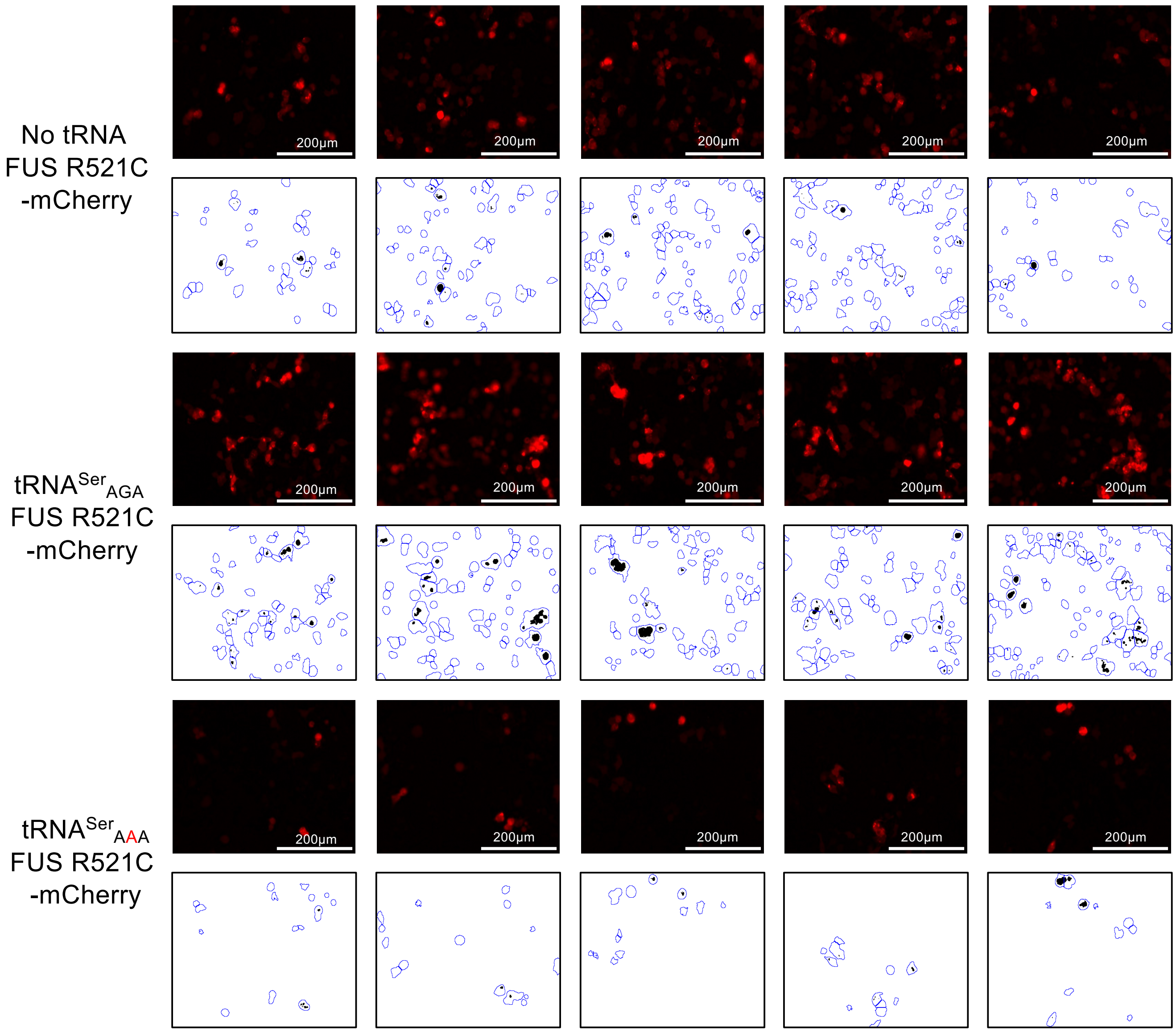

Figure S8. Additional images of FUS R521C-mCherry aggregates in N2a cells. N2a cells were transfected with a plasmid encoding no tRNA (top), human $tRNA^{Ser}_{AGA}$ (middle), or $tRNA^{Ser}_{AAA}$ (bottom) and FUS R521C-mCherry**.** Representative images (see also Figs. 6) of fluorescing cells were captured by live-cell fluorescence microscopy (RFP ex. 531 nm, em. 593 nm) beginning 25 hours after transfection for a 43.5-hour time course. Fluorescent cell area representing cells and aggregates was determined using a custom Fiji/ImageJ macro. Cell and aggregate counts were determined based on the observed median cells and aggregate area (see supplementary methods and appendix). Representative images of the fluorescing cells (above) and black and white mask images (below) showing aggregate area (black) and cell area (blue outlines).

S.5 SUPPLEMENTARY VIDEO CAPTIONS

Supplementary Video 1. The supplementary video file shows representative fluorescence microscopy images of the time course of FUS aggregation kinetics shown in Fig. 5. Videos representing cells that co-express FUS-mCherry or FUS R521C-mCherry and no additional tRNA, $tRNA^{Ser}_{AGA}$, or $tRNA^{Ser}_{AAA}$. Cell rupture events identified in mistranslating cells co-expressing FUS R521C are annotated (white arrow).

Supplementary Video 2. The supplementary video file shows additional representative fluorescence microscopy images of the time course of FUS R521C aggregation kinetics shown in Fig. 5. Videos represent cells that co-express FUS R521C-mCherry and no additional tRNA, $tRNA^{Ser}_{AGA}$, or $tRNA^{Ser}_{AAA}$. Cell rupture events identified in cells expressing FUS R521C are annotated (white arrow).

S.6 SUPPLEMENTARY DATA FILE S1

Excel spreadsheet containing raw read counts for tRNA sequencing of N2a cells expressing $tRNA^{Ser}_{AGA}$ or $tRNA^{Ser}_{AAA}$.

S.7 SUPPLEMENTARY APPENDIX

Fiji/ImageJ macros. ImageJ (version 1.53f51 [85]; Java version 1.8.0_172 (64-bit)) scripts to measure the fluorescence and analyze images of transfected cells. All macros were run on 16-bit, single-channel images. Lines of commentary in the macros are denoted with "//."

Fiji/ImageJ macros used in Figure 1, 3C, 5, S4 – Mean fluorescence per cell plots. Fluorescence in regions of interest (ROI; fluorescing cells) was measured with the following Macro in Fiji/ImageJ. Since some ROIs capture multiple cells in clusters, ROI areas were weighted in Microsoft Excel by dividing the area of each ROI by median ROI size (i.e., median cell size) for the entire experiment. Hence, all fluorescence measurements underlying the means were weighted to the same fixed area, which approximates median cell size. Standard deviations were calculated based on the means of at least N = 3 biological replicates at each timepoint.

```
//Import image sequence. Replace "folder name" with desired image series location. "number" is the
number of images in the series and "increment" is used to exclude unwanted images in the file folder.
run("Image Sequence...", "open=["folder name"] number=88 starting=5 increment=3 sort");
//Subtract background signal using rolling circle method with radius of 50 pixels.
run("Subtract Background...", "rolling=50 stack");
//Set threshold to exclude pixels below chosen cutoff (6 in this case). Fine-tune according to your image
brightness and use same cutoff for all images in one experiment.
setThreshold(6, 255);
//Next two lines convert image to black features (above threshold) on white background (below
threshold)
setOption("BlackBackground", false);
run("Convert to Mask", "method=Default background=Dark");
//Remove noise with despeckle function.
run("Despeckle", "stack");
```

//Create annotations covering area above threshold. Excludes objects of area under chosen size cut-off (20 pixels in this case). Size cut-off should be adjusted depending on the magnification of the image and the size of objects you wish to exclude (noise).
run("Analyze Particles...", "size=20-Infinity display clear summarize add stack");
//Clear results. Steps so far to are to create region of interest annotations (ROIs), results will be collected in following steps.
run("Clear Results");
//Import unmodified image sequence again. Should be identical to line 1
run("Image Sequence...", "open=[“folder name”] number=88 starting=5 increment=3 sort");
//Subtract background signal using rolling circle method with radius of 50 pixels.
run("Subtract Background...", "rolling=50 stack");
//Set measurements to record area, mean, integrated density to 3 decimal places.
run("Set Measurements...", "area mean integrated redirect=None decimal=3");
//The next three lines overlay the ROIs on the fluorescent cell image sequence, calculate the intensity within the ROIs, and display the results in the “Results” window.
roiManager("Show None");
roiManager("Show All");
roiManager("Measure");

Fiji/ImageJ macros used in Figure 3D, 6, S6 – Number of aggregates per cell plots. The same ROIs (outlining whole cell area) used in Figure 2 fluorescence analysis were used for cell size determination. To determine the size of aggregates, black and white mask images were produced at a higher intensity threshold to select for the brightest foci emanated from protein aggregates. We followed established methods [17,49] to define a threshold to count intensely bright foci as protein aggregates. The mean cellular fluorescence for all FUS-mCherry or FUS(R521C)-mCherry cell lines was 7.45 units/pixel (background subtracted and scaled from 0-255), and the standard deviation was 5.02 units/pixel. The threshold to define aggregated protein foci was then set to 100 units/pixel, which represents 18 standard deviations above the mean fluorescent pixel value. Then the whole cell ROIs were overlaid on the aggregate mask image and “area” and “area fraction” were measured. Aggregate area per ROI was calculated in Microsoft Excel as the product of (Area · Area Fraction). Since some ROIs capture multiple cells or aggregates in clusters, cell and aggregate areas were weighted in Microsoft Excel by dividing the area of each ROI by median cell or aggregate size calculated over the entire experiment. Standard deviation was calculated based on the means of at least N = 3 biological replicates at each timepoint.

//Import image sequence. Replace “folder name” with desired image series location. “number” is the number of images in the series and “increment” is used to exclude unwanted images in the file folder.
run("Image Sequence...", "open=[“folder name”] number=88 starting=5 increment=3 sort");
//Subtract background signal using rolling circle method with radius of 50 pixels.
run("Subtract Background...", "rolling=50 stack");
//Set threshold to exclude pixels below chosen cutoff (6 in this case). Fine-tune according to your image brightness and use same cutoff for all images in one experiment. In this step, threshold should be adjusted to capture whole cell area.
setThreshold(6, 255);
//Next two lines convert image to black features (above threshold) on white background (below threshold).
setOption("BlackBackground", false);

```
run("Convert to Mask", "method=Default background=Dark");
//Remove noise with despeckle function.
run("Despeckle", "stack");
//Separate closely adjacent objects with watershed function.
run("Watershed", "stack");
//Create annotations covering area above threshold. Excludes objects of area under chosen size cut-off (100 pixels in this case). Size cut-off should be adjusted depending on the magnification of the image and the size of objects you wish to exclude (noise).
run("Analyze Particles...", "size=100-Infinity display clear summarize add stack");
//Clear results. Steps so far to are to create region of interest annotations (ROIs), results will be collected in following steps.
run("Clear Results");
//Import unmodified image sequence again. Should be identical to line 1
run("Image Sequence...", "open=[“folder name”] number=88 starting=5 increment=3 sort");
//Subtract background signal using rolling circle method with radius of 50 pixels.
run("Subtract Background...", "rolling=50 stack");
//Set threshold to exclude pixels below chosen cutoff (100 in this case). Fine-tune according to your image brightness and use same cutoff for all images in one experiment. In this step, threshold should be adjusted to capture protein aggregate area only.
setThreshold(100, 255);
//Convert image to black features (above threshold) on white background (below threshold).
run("Convert to Mask", "method=Default background=Dark");
//The next two lines overlay the ROIs from whole cell threshold on the mask image produced from the aggregate threshold.
roiManager("Show None");
roiManager("Show All");
//Set measurements to record area and area fraction to 3 decimal places. Area will output the whole cell ROI areas, area fraction will output the fraction of that area containing an aggregate object from the aggregate mask image. Aggregate area can then be calculated as the product of area x area fraction.
run("Set Measurements...", "area area_fraction redirect=None decimal=3");
//Measure.
roiManager("Measure");
```

Fiji/ImageJ macros used in Figure S5 – Number of aggregates per total fluorescence. Figure S3 was analyzed with a previously described ImageJ macro [17]. A lower threshold was used to generate ROIs capturing all fluorescence in each image (A, cellular fluorescence) and measure the total fluorescence in each image in the series. A higher threshold was used to generate ROIs capturing only aggregated FUS-EGFP protein (B, aggregate area). The mean cellular fluorescence for all FUS-EGFP cell lines was 5.69 units/pixel (background subtracted and scaled from 0-255), and the standard deviation was 6.73 units/pixel. The threshold to define aggregated protein foci was then set to 120 units/pixel, which represents 17 standard deviations above the mean fluorescent pixel value. Total fluorescence and aggregate area were calculated per image in Microsoft Excel using the SUMIF() function. For each image, total aggregate area was normalized to total transfected cell fluorescence and initial values were subtracted from all datapoints in the series to obtain “Δ $N_{aggregates}$ / total fluorescence”. Standard deviation was calculated based on the means of at least three biological replicates at each timepoint.

A) Cellular fluorescence

//Import image sequence. Replace “folder name” with desired image series location. “number” is the number of images in the series and “increment” is used to exclude unwanted images in the file folder.
run("Image Sequence...", "open=[“folder name”] number=37 starting=2 increment=3 sort");
//Subtract background signal using rolling circle method with radius of 50 pixels.
run("Subtract Background...", "rolling=50 stack");
//Set threshold to exclude pixels below chosen cutoff (6 in this case). Fine-tune according to your image brightness and use same cutoff for all images in one experiment. In this step, threshold should be adjusted to capture whole cell area.
setThreshold(6, 255);
//Next two lines convert image to black features (above threshold) on white background (below threshold).
setOption("BlackBackground", false);
run("Convert to Mask", "method=Default background=Dark");
//Remove noise with despeckle function.
run("Despeckle", "stack");
//Create annotations covering area above threshold. Excludes objects of area under chosen size cut-off (20 pixels in this case). Size cut-off should be adjusted depending on the magnification of the image and the size of objects you wish to exclude (noise).
run("Analyze Particles...", "size=20-Infinity display clear summarize add stack");
//Clear results. Steps so far to are to create region of interest annotations (ROIs), results will be collected in following steps.
run("Clear Results");
//Import unmodified image sequence again. Should be identical to line 1
run("Image Sequence...", "open=[“folder name”] number=37 starting=2 increment=3 sort");
//Subtract background signal using rolling circle method with radius of 50 pixels.
run("Subtract Background...", "rolling=50 stack");
//The next two lines overlay the ROIs from the thresholded on the non-thresholded image.
roiManager("Show None");
roiManager("Show All");
//Set measurements to record integrated density to 3 decimal places, then measure.
run("Set Measurements...", "integrated redirect=None decimal=3");
roiManager("Measure");

B) Aggregate area

//Import image sequence. Replace “folder name” with desired image series location. “number” is the number of images in the series and “increment” is used to exclude unwanted images in the file folder.
run("Image Sequence...", "open=[“folder name”] number=37 starting=2 increment=3 sort");
//Subtract background signal using rolling circle method with radius of 50 pixels.
run("Subtract Background...", "rolling=50 stack");
//Set threshold to exclude pixels below chosen cutoff (120 in this case). Fine-tune according to your image brightness and use same cutoff for all images in one experiment. In this step, threshold should be adjusted to capture aggregated protein area.
setThreshold(120, 255);
//Next two lines convert image to black features (above threshold) on white background (below threshold).

```
setOption("BlackBackground", false);
run("Convert to Mask", "method=Default background=Dark");
//Create annotations covering area above threshold. Excludes objects of area under chosen size cut-off (5 pixels in this case). Size cut-off should be adjusted depending on the magnification of the image and the size of objects you wish to exclude (noise).
run("Analyze Particles...", "size=5-Infinity display clear summarize add stack");
//Set measurements to record area to 3 decimal places, then measure.
run("Set Measurements...", "area redirect=None decimal=3");
roiManager("Measure");
```
